# Metalloporphyrin-based hybrid photocatalyst for bisphenol A degradation: kinetics, HRMS-based analysis of transformation products, and toxicity assessment

**DOI:** 10.1039/d6ra01469k

**Published:** 2026-04-13

**Authors:** Christina-Konstantina Tsamtzidou, Athanasios Theodoridis, Dimitrios Rafail Bitsos, Michail Chalaris, Kalliopi Ladomenou, Christina Nannou

**Affiliations:** a Hephaestus Laboratory, School of Chemistry, Faculty of Sciences, Democritus University of Thrace GR-65404 Kavala Greece nannou@chem.duth.gr kladomenou@chem.duth.gr

## Abstract

Bisphenol A (BPA) is a persistent endocrine-disrupting compound frequently detected in aquatic environments and inadequately removed by conventional wastewater treatment processes. In this work, a visible-light-active TiO_2_-metalloporphyrin hybrid photocatalyst was developed *via* surface sensitization of TiO_2_ with zinc tetrakis(4-carboxyphenyl)porphyrin (ZnTCPP) and evaluated for BPA degradation in aqueous media. The hybrid material was characterized by UV-Vis spectroscopy (UV-Vis), Fourier Transform Infrared (FT-IR) Spectroscopy, powder X-ray diffraction (PXRD), and scanning electron microscopy (SEM), confirming successful porphyrin immobilization without altering the anatase crystal structure. Photocatalytic experiments under visible-light irradiation demonstrated efficient BPA removal, reaching up to 70% degradation within 180 min, while maintaining stable performance over three successive cycles. Kinetic analysis revealed that BPA degradation followed a power law kinetic model, with enhanced reaction rates under acidic conditions and optimized catalyst loading. Radical scavenging experiments indicated that superoxide and hydroxyl radicals were the dominant reactive species governing the oxidation process. High-resolution Orbitrap mass spectrometry enabled the identification of major transformation products and the elucidation of degradation pathways, which were dominated by aromatic hydroxylation and C–C bond cleavage reactions. *In silico* ECOSAR toxicity assessment showed a substantial reduction in acute and chronic aquatic toxicity for the main intermediates compared with the parent BPA molecule. Overall, the TiO_2_-metalloporphyrin hybrid effectively extends photocatalytic activity into the visible region and enables efficient pollutant removal with reduced environmental risk, highlighting its potential for sustainable solar-driven water treatment applications.

## Abstract 2

ZnTCPP-TiO_2_ photocatalytic degradation of bisphenol A: kinetics, elucidation of transformation products, and toxicity assessment.

## Graphical abstract

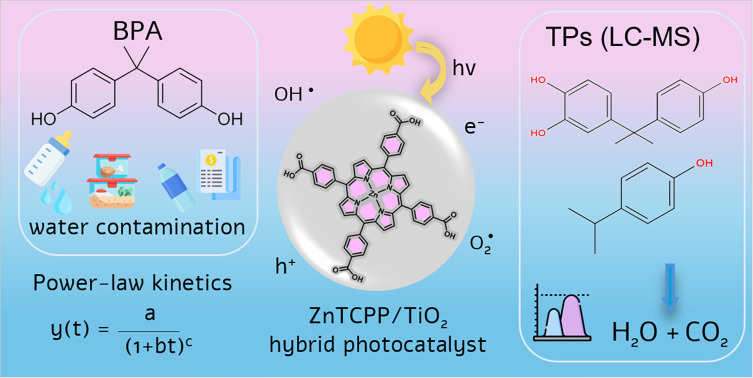

## 1 Introduction

Bisphenol A (BPA, 4,4′-isopropylidenediphenol) is a high-production-volume chemical primarily used in the manufacture of polycarbonate plastics and epoxy resins, with additional applications in optical and electronic materials.^1,2^ Its extensive use in consumer products, such as food-contact materials, water pipes, and thermal paper, has led to its widespread release into the environment and frequent detection in surface waters and wastewater effluents, typically at ng L^−1^ to µg L^−1^ levels.^3^ Owing to its persistence and continuous input, BPA is now regarded as a ubiquitous aquatic contaminant of particular concern.^4–6^

Beyond its environmental occurrence, BPA is recognized as a potent endocrine-disrupting chemical (EDC) with estrogen-mimicking activity, capable of interfering with reproductive, neurological, and immune functions.^7^ These concerns have led to its classification as a Substance of Very High Concern (SVHC) under the EU REACH framework due to its reproductive toxicity and endocrine-disrupting properties. Biomonitoring studies across Europe have further demonstrated that more than 90% of the adult population exhibits detectable BPA levels in urine, indicating chronic human exposure.^8,9^ In response to emerging toxicological evidence, the European Food Safety Authority (EFSA) revised the tolerable daily intake (TDI) of BPA in 2023 from 4 µg kg^−1^ body weight per day to a highly restrictive 0.2 ng kg^−1^ body weight per day, reflecting the compound's adverse immunomodulatory effects at ultra-low exposure levels.^10^ This regulatory reassessment was followed by the introduction of a comprehensive ban on BPA and related bisphenols in food-contact materials within the European Union, which came into force in 2024.^1,11^

Although regulatory restrictions have significantly limited the production and use of BPA, its continuous release from existing materials and its environmental persistence render conventional mitigation strategies insufficient. Standard biological wastewater treatment processes exhibit limited removal efficiency for BPA, while partial degradation often leads to the formation of transformation products that may retain endocrine-disrupting activity.^12^ Consequently, the development of advanced treatment technologies capable of achieving deep oxidation and detoxification has become a priority.^13^ Advanced oxidation processes (AOPs) have emerged as particularly promising approaches for the removal of organic contaminants due to their ability to generate highly reactive oxygen species, such as hydroxyl radicals and superoxide anions, which can non-selectively oxidize persistent pollutants.^14^

Among these technologies, semiconductor photocatalysis has attracted considerable attention owing to its chemical stability, operational simplicity and potential to utilize solar energy as a sustainable driving force.^15–17^ Titanium dioxide (TiO_2_) remains the most widely studied photocatalyst because of its strong oxidative power, low cost and environmental compatibility.^18,19^ Upon photoexcitation, TiO_2_ generates electron–hole pairs that initiate the formation of reactive oxygen species capable of mineralizing organic pollutants, including endocrine-disrupting compounds such as BPA.^20^ Despite its widespread use and favorable properties, the practical application of TiO_2_ photocatalysis is hindered by intrinsic limitations.^19^ The wide bandgap of anatase TiO_2_ (3.2 eV) restricts its photoactivity primarily to the ultraviolet region of the solar spectrum, which represents less than 5% of the total solar irradiance. In addition, the rapid recombination of photogenerated electron–hole pairs diminishes its quantum efficiency.^19,21^ To address these challenges, various approaches have been proposed, including metal and non-metal doping, coupling with narrow-bandgap semiconductors, plasmonic metal decoration and surface sensitization with organic chromophores.^22,23^ Among these strategies, dye sensitization using porphyrins and metalloporphyrins offers distinct advantages due to their strong absorption in the visible region, tunable electronic structure and structural similarity to natural light-harvesting systems.^24–26^ Porphyrin macrocycles exhibit intense Soret and Q bands, enabling efficient utilization of solar photons, while peripheral functional groups allow stable anchoring onto metal oxide surfaces and promote electron transfer.^27,28^ When immobilized on TiO_2_, metalloporphyrins act as molecular antennas that harvest visible light and inject excited electrons into the conduction band of the semiconductor, thereby extending photoactivity into the visible spectrum.^29,30^ This dye-sensitized mechanism not only enhances photon utilization but also improves spatial charge separation, suppressing recombination and increasing reactive oxygen species generation.^31^ As a result, porphyrin-sensitized TiO_2_ systems have demonstrated improved performance in the degradation of persistent organic contaminants under visible light.^32^

In this context, the present study reports the design and evaluation of a TiO_2_-metalloporphyrin hybrid photocatalyst for the visible-light-driven degradation of bisphenol A in aqueous media. The hybrid material was prepared *via* surface sensitization of TiO_2_ with zinc tetrakis(4-carboxyphenyl)porphyrin (ZnTCPP), enabling strong visible-light absorption and efficient interfacial charge transfer. Photocatalytic performance was systematically investigated under different conditions, including pH, catalyst loading, pollutant concentration, and the presence of radical scavengers. In addition to assessing degradation efficiency and reaction kinetics, high-resolution Orbitrap mass spectrometry was employed to elucidate transformation pathways and identify major intermediates formed during BPA oxidation. Finally, the environmental relevance of the treatment was evaluated through *in silico* ECOSAR toxicity modeling, allowing assessment of potential ecological risks associated with the generated transformation products. By integrating photocatalytic performance, mechanistic insight and toxicity evaluation, this work provides a comprehensive assessment of the environmental applicability of metalloporphyrin-sensitized TiO_2_ systems and highlights their potential as sustainable, visible-light-active platforms for the removal of endocrine-disrupting contaminants from water.

## 2 Experimental

### 2.1 Materials and methods

All chemicals were purchased from certified suppliers and used as received. The materials for the synthesis of the ZnTCPP porphyrin (Zinc Acetate, Propionic Acid, Dichloromethane, Methanol, Pyrrole, and Methyl 4-formylbenzoate) were purchased from Sigma Aldrich (≥99.9% synthesis reagents). TiO_2_ anatase nanoparticles for the synthesis of the hybrid material (TiO_2_-ZnTCPP) were also purchased from Sigma-Aldrich. The metalloporphyrins were synthesized according to previously reported literature procedures.^33,34^ The porphyrin moieties were analyzed with ^1^H NMR spectroscopy using Bruker AMX-500 MHz. Bruker ultrafleXtreme Matrix Assisted Laser Desorption Ionization (MALDI) time-of-flight mass spectrometer was employed for recording MALDI-TOF mass spectra. Samples were dissolved in the appropriate solvent and mixed with a matrix (*trans*-2-[3-(4-*tert*-butylphenyl)-2-methyl-2-propenylidene]) when necessary. A white LED Lamp 100 W (Visible Light) was employed for the photocatalysis experiments.

Bisphenol A (BPA ≥99% purity, CAS No. 80-05-7) was purchased from Merck (Darmstadt, Germany). Stock solutions of the model compound were prepared in ultrapure water and stored at −20 °C, whereas fresh working solutions were prepared daily before each photocatalytic treatment. The selected initial concentration was greater than what is typically found in waters/wastewaters and was purposefully defined to facilitate the elucidation of transformation products (TPs). Additional reagents, such as hydrogen peroxide (H_2_O_2_) 30% (w/w) in H_2_O, and buffers were purchased from Sigma-Aldrich. LC-MS/MS grade solvents (acetonitrile, methanol, isopropanol, and water) were purchased from Merck (Darmstadt, Germany). Formic acid (HCOOH) of LC-MS grade (98%) was obtained from Sigma Aldrich (Germany). Ultrapure water was obtained using a purification system (18.2 MΩ × cm, Milli-Q, Millipore, USA). Spectrophotometric measurements were performed using a UV-Vis spectrometer (UV/Vis uniSPEC 4, LLG-Labware, Meckenheim, Germany), whereas the photocatalytic experiments utilized a lab-made solar simulator (white LED lamp, 100 W). High-resolution mass spectrometry was conducted using an Orbitrap™ high-resolution mass spectrometer (Thermo Fisher Scientific) coupled with an Accela™ ultra-high-pressure liquid chromatography system. All glassware was rinsed with Milli-Q water before use. Experiments were performed in triplicate to ensure reproducibility.

### 2.2 Synthesis of the hybrid TiO_2_-metalloporphyrin

The TiO_2_-metalloporphyrin hybrid material (TiO_2_-MP) was synthesized by adsorbing ZnTCPP onto TiO_2_ using a dye-sensitization protocols, adapted from previously reported work.^34^ A concentrated stock solution of the metalated porphyrin was first prepared in tetrahydrofuran (THF) and subsequently diluted to obtain six solutions with concentrations of 1 × 10^−5^, 5 × 10^−5^, 10 × 10^−5^, 15 × 10^−5^, 20 × 10^−5^ and 30 × 10^−5^ mol L^−1^ (Fig. S1).

UV-Vis absorption spectra of the porphyrin solutions were recorded in the 400–800 nm range to determine the initial absorbance of the characteristic Soret and Q bands. Subsequently, 5 mg of TiO_2_ was added to each solution, and the resulting suspensions were sonicated for 5 min and magnetically stirred in the dark for 40 min to ensure adsorption equilibrium. Following the adsorption process, the suspensions were centrifuged at 2000 rpm for 20 min. The supernatants were collected and analyzed by UV-Vis spectroscopy in the same spectral range. The decrease in porphyrin absorbance was used to quantify the amount of ZnTCPP adsorbed onto the TiO_2_ surface according to established methodologies.^35^ On the basis of adsorption efficiency, a porphyrin concentration of 5 × 10^−5^ mol L^−1^ was selected as optimal for the sensitization process (Fig. S2). The resulting TiO_2_-ZnTCPP hybrid photocatalyst was then recovered, thoroughly dried, and used for subsequent physicochemical characterization and photocatalytic performance evaluation.

### 2.3 Characterization of the hybrid TiO_2_-metalloporphyrin

The porphyrin intermediates TPPCOOMe, ZnTPPCOOMe, and ZnTCPP were characterized by UV-Vis and Fourier-transform infrared (FT-IR) spectroscopy to confirm successful metalation and ester hydrolysis. UV-Vis spectra were recorded in appropriate organic solvents to monitor the characteristic Soret and Q bands of the porphyrin macrocycle. FT-IR spectra were collected in the 4000–400 cm^−1^ region, enabling the identification of the main vibrational modes associated with the porphyrin core and peripheral functional groups. Structural information of the TiO_2_ and TiO_2_-MP hybrid material was obtained using powder X-ray diffraction (PXRD) in the 2*θ* range of 5–80°, enabling the identification of the crystalline phases present in the samples. In addition, UV-Vis absorption spectra in the 400–800 nm range were recorded for TiO_2_-ZnTCPP dispersions during the photocatalytic degradation experiments using bisphenol A (BPA). These measurements were used to monitor the evolution of BPA concentration over time and to evaluate the photocatalytic performance of the hybrid material under visible-light irradiation.

### 2.4 Photocatalytic experiment and kinetic study

**Fig. 1:**
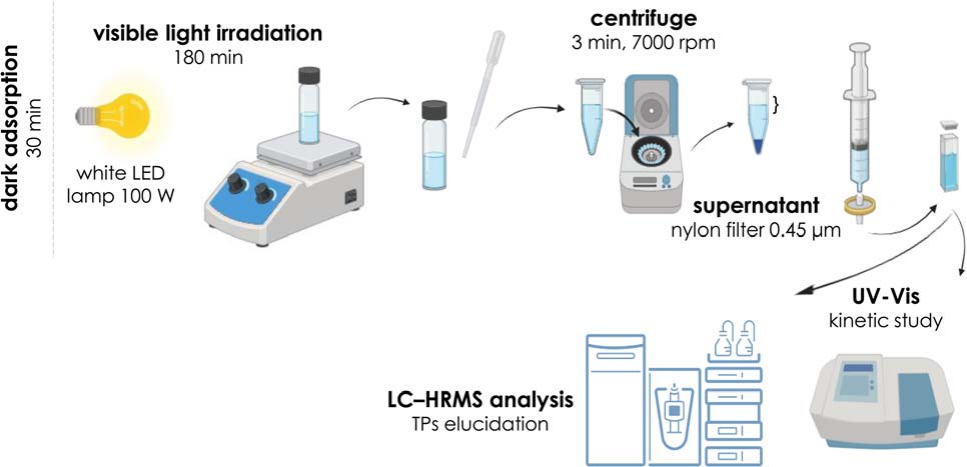
Schematic illustration of the experimental set-up.

Photocatalytic experiments were conducted in a custom-built reactor equipped with a 100 W white LED lamp under continuous magnetic stirring (Fig. 1). The reaction temperature was maintained at 25 °C. An appropriate volume of BPA solution (10 mL) was added to the reactor, followed by the addition of the desired amount of catalyst. Before illumination, the suspension was kept in the dark for 30 min under stirring to establish adsorption–desorption equilibrium between BPA and the catalyst surface. After this equilibration period, the light source was switched on, and aliquots were withdrawn at predefined time intervals. The collected samples were centrifuged and analyzed by UV-Vis spectroscopy. Samples intended for high-resolution mass spectrometry (HRMS) analysis, including scavenger studies and TP identification, were additionally filtered through 0.22 µm nylon syringe filters prior to analysis.

### 2.5 Identification of TPs

The transformation products were identified using an LC-Orbitrap MS/MS system consisting of a hybrid LTQ-FT Orbitrap XL 2.5.5 SP1 mass spectrometer (Thermo Fisher Scientific, Bremen, Germany). The linear ion trap (LTQ) of the system included ion maximum electrospray ionization (ESI) operating in the negative ionization mode. The MS system was coupled with an Accela UHPLC equipped with an autosampler (model 2.1.1) and binary gradient pump (model 1.05.0900). The separation of the analytes was achieved on a Thermo Hypersil GOLD column (50 × 2.1 mm, 2.6 µm), kept at 40 °C throughout the analysis. The injection volume was set at 20 µL and the flow rate was stable at 0.3 mL min^−1^. The duration of the gradient elution program was 15 min using ammonium formate 5 mM to acidify the mobile phase (water and methanol). More information on the LC conditions, MS parameters, and data treatment workflow is provided in SI (Table S1 and S2).

BPA and its major TPs were identified as [M − H]^−^ pseudo-molecular ions. The mass range selected for full scan acquisition was *m*/*z* 120–400 amu at a resolution of 60 000 FWHM, while the fragments were recorded at 15 000 FWHM resolution. A normalized collision energy (NCE) of 35% was employed to ensure identification based on the fragmentation patterns of the analytes. MS/MS identification of TPs was guided by fragmentation patterns accompanied by potential structures created using MolView (https://app.molview.com/). Time-resolved sampling enabled the profiling of degradation pathways and major intermediates. Raw data obtained from LC-HRMS were acquired and processed using XCalibur v2.2 (Thermo Scientific). A kinetic study was performed using Gnuplot.

### 2.6 *In silico* toxicity assessment

In accordance with the REACH regulation,^36^ the toxicity of BPA and its major TPs was estimated individually *in silico,* with the Ecological Structure Activity Relationships (ECOSAR) software (US EPA, v2.2), a common tool used for the prediction of acute and chronic aquatic toxicity (LC50, EC50, ChV) for fish, daphnids, and algae. The suggested structure for each TP was inserted into the software with the aid of Simplified Molecular Input Line Entry System (SMILES) strings. The results were interpreted considering EU environmental safety limits (Regulation No. 1272/2008/EC).^37^ Four toxicity levels were considered to evaluate the extent of potential toxicity: (i) values ranging from 0.0 to 1.0, very toxic; (ii) values from 1 to 10, toxic; (iii) values from 10 to 100, harmful effects; and (iv) values > 100, non-toxic.

## 3 Results and discussion

### 3.1 Characterization of the photocatalyst

#### 3.1.1 Synthesis and UV-Vis spectra of ZnTCPP

**Fig. 2:**
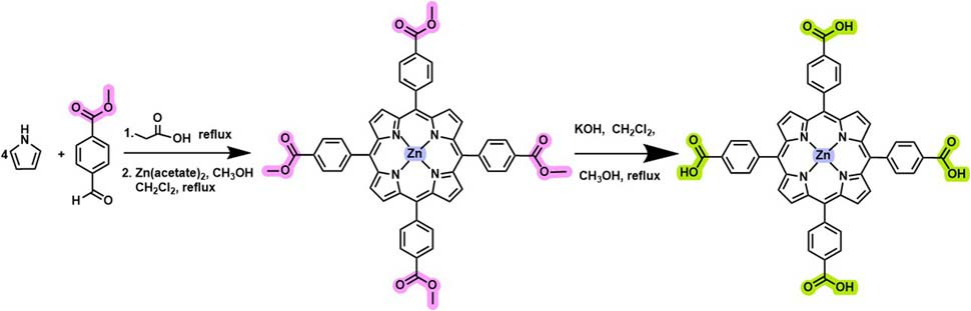
Synthetic route for the preparation of ZnTCPP.

The three-step synthesis of ZnTCPP (Fig. 2), involving porphyrin formation (TPPCOOMe), zinc metalation (ZnTPPCOOMe), and subsequent ester hydrolysis, was successfully achieved with good overall yields. Initially, the condensation of 4-formylbenzoate methyl ester (3 g) with pyrrole (1.4 mL) in refluxing propionic acid (100 mL) for 3 h afforded TPPCOOMe in 20.7% yield after purification by recrystallization using a dichloromethane/methanol (CH_2_Cl_2_/MeOH) solvent system, in agreement with previously reported methods.^38^ Metallation of TPPCOOMe (300 mg) was achieved by reaction with zinc acetate (750 mg) in a CH_2_Cl_2_/MeOH mixture (20 : 3, v/v) under reflux for 2 h, yielding ZnTPPCOOMe in 76.9% yield. Residual free-base porphyrin was efficiently removed by column chromatography using 1% MeOH in CH_2_Cl_2_ as the eluent. Final hydrolysis of the ester groups was performed by treating ZnTPPCOOMe (50 mg) with KOH in THF/MeOH (2 : 1, v/v) under reflux for 12 h, resulting in quantitative conversion to ZnTCPP (100% yield). UV-Vis absorption spectroscopy was employed to monitor the successful progression of each synthetic step. The spectrum of TPPCOOMe displayed a strong Soret band at 420 nm and four Q bands at 515, 550, 590, and 646 nm, which are characteristic of free-base tetraarylporphyrins, and consistent with literature reports (Fig. S3). Upon zinc insertion, the four Q bands collapsed into two, reflecting the increased symmetry of the metalloporphyrin macrocycle and confirming the successful metalation from TPPCOOMe to ZnTPPCOOMe (Fig. S4).^39^ Subsequent hydrolysis of the methyl ester groups to yield ZnTCPP resulted in increased macrocycle polarity and induced a noticeable bathochromic shift, particularly in the Q-band region of the absorption spectrum (Fig. S5). This red shift is attributed to the introduction of electron-withdrawing carboxylic acid groups, which modify the electronic distribution of the macrocycle. Importantly, this spectral shift enhances visible-light absorption and facilitates surface anchoring to TiO_2_, both of which are beneficial for the performance of the hybrid photocatalyst.^40^

#### 3.1.2 ^1^H NMR and MALDI-TOF-MS analysis of TPPCOOMe and ZnTCPP

The structure and purity of the free-base porphyrin precursor TPPCOOMe were confirmed by ^1^H NMR and MALDI-TOF-MS analysis (Fig. S9 and S10). The β-pyrrolic protons appear as a set of signals in the aromatic region (*δ* 8.84 ppm), while the phenyl protons of the p-substituted aryl rings give rise to the expected two doublet of doublets between *δ* = 8.48, 8.46 and 8.33, 8.31 ppm. The methoxy groups of the ester functions resonate as a sharp singlet near *δ* = 4.14 ppm, confirming the presence of four methyl ester substituents. The overall integration pattern and the absence of additional resonances indicate a single, well-defined porphyrinic species with high purity.^41,42^

MALDI-TOF-MS further corroborates the molecular structure of TPPCOOMe (Fig. S7). The mass spectrum exhibits an intense molecular ions peak at *m*/*z* = 846.51, in excellent agreement with the calculated mass for TPPCOOMe (*m*/*z* = 846.89), with no significant satellite peaks attributable to demetallated, oxidized, or partially substituted derivatives. The close match between the experimental and theoretical molecular weights, together with the clean isotopic pattern (844.65–849.52), confirms the successful synthesis of TPPCOOMe and provides a robust basis for the subsequent metalation and hydrolysis steps leading to ZnTCPP.^41,42^

The structure of the metalated porphyrin ZnTCPP was further verified by ^1^H NMR spectroscopy in *d*_6_-DMSO and MALDI-TOF-MS (Fig. S11 and S12). The ^1^H NMR spectrum of ZnTCPP (Fig. S8) shows the expected disappearance of the inner N–H singlet observed for TPPCOOMe, consistent with full coordination of Zn^2+^ at the porphyrin core. The β-pyrrolic resonances remain in the aromatic region (*δ* ≈ 8.8 ppm), while the para-substituted phenyl protons again appear as two sets of doublets, confirming preservation of the tetraaryl porphyrin framework. In contrast to the ester precursor, the methoxy singlet at *δ* ≈ 4.1 ppm is absent, and the spectrum displays broadened signals attributable to the carboxylic acid/carboxylate substituents on the phenyl rings, in agreement with complete hydrolysis of the –COOCH_3_ groups to –COOH. The clean proton pattern and lack of extraneous peaks indicate a single metallated species of high purity.^43^

MALDI-TOF-MS analysis of ZnTCPP (Fig. S9) exhibits a dominant molecular ion peak at the expected *m*/*z* value for ZnTCPP (*m*/*z* = 851.29), in excellent agreement with the calculated mass, accompanied by a well-resolved isotopic distribution characteristic of a Zn-containing porphyrin. The absence of significant additional peaks corresponding to demetallated, partially hydrolysed, or oligomeric species confirms that the metalation and ester hydrolysis steps proceed cleanly to the desired ZnTCPP product. Together with the UV-Vis and FT-IR data, these NMR and MALDI-TOF-MS results provide comprehensive confirmation of the ZnTCPP structure used for TiO_2_ sensitization.^43^

#### 3.1.3 FT-IR spectra

FT-IR spectroscopy was used to further confirm the successive structural changes occurring during the synthesis of ZnTCPP (Fig. S10). The spectrum of TPPCOOMe, displayed a characteristic absorption band at 3316 cm^−1^, attributed to N–H stretching vibrations of the free-base porphyrin core. Bands in the 3000–3100 cm^−1^ region, corresponded to C–H stretching of sp^2^-hybridized carbon atoms, while additional features between 2800 and 3000 cm^−1^ range were assigned to sp^3^ C–H stretching vibrations. Weak absorptions observed in the 1800–2000 cm^−1^ region were associated with aromatic C–H overtone and combination bands. A strong band at 1712 cm^−1^ confirmed the presence of ester carbonyl (C=O) groups in the TPPCOMe. This was accompanied by bands at approximately 1601 cm^−1^, assigned to aromatic C=C stretching vibrations, as well as peaks at 1265 and 1097 cm^−1^, corresponding to C–O stretching vibrations of the ester functionality. Following zinc metalation, the disappearance of the N–H stretching band at 3316 cm^−1^ provided clear evidence for the successful coordination of Zn^2+^ into the porphyrin core (Fig. S11). Importantly, the ester-related C=O and C–O stretching bands remained essentially unchanged, indicating that metal insertion occurred selectively at the macrocyclic center without affecting the peripheral ester substituents. Subsequent hydrolysis of ZnTPPCOOMe to yield ZnTCPP led to pronounced spectral changes. In particular, the ester carbonyl band at ∼1712 cm^−1^ disappeared, while new absorption features associated with carboxylic acid and/or carboxylate groups emerged. These changes were accompanied by modifications in the fingerprint region, consistent with the conversion of –COOCH_3_ moieties to –COOH groups on the phenyl rings (Fig. S12). The observed FT-IR features were in good agreement with previously reported data for carboxyl-functionalized zinc porphyrins and related hybrid materials.^44–46^

#### 3.1.4 PXRD of TiO_2_ and TiO_2_-MP

**Fig. 3:**
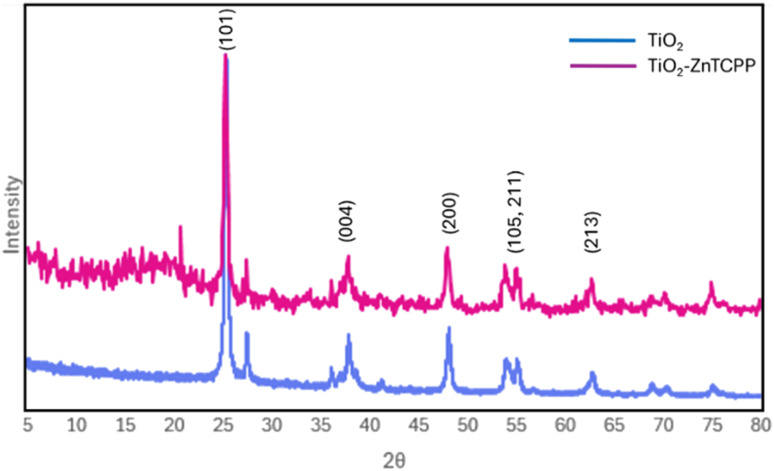
PXRD spectra of pure TiO_2_ (blue) and TiO_2_-ZnTCPP (purple).

Powder X-ray diffraction (PXRD) patterns of commercial anatase TiO_2_ and the TiO_2_-ZnTCPP hybrid photocatalyst were recorded in the 2*θ* range of 5–80° (Fig. 3). The diffraction pattern of pristine TiO_2_ (blue) exhibited characteristic reflections of the anatase phase at 25.3° (101), 37.9° (004), 48.1° (200), 55.1° [(105), (211)], and 62.7° (213), which is in good agreement with the standard reference data. Importantly, the TiO_2_-ZnTCPP hybrid(purple) displayed identical peak positions and relative intensities for these reflections, indicating that the adsorption of ZnTCPP did not perturb the bulk crystal structure of TiO_2_ support. This preservation of the anatase phase is essential, as it ensures that the intrinsic semiconductor properties required for efficient photocatalytic activity remain intact.

In addition to the anatase reflections, the TiO_2_-ZnTCPP hybrid displayed distinct low-angle features that were absent in pristine TiO_2_. Specifically, a slight elevation of the diffraction baseline below 20° 2*θ* and a weak but sharp peak cantered at approximately 20° 2*θ* was observed. These features are attributed to partial ordering or layered stacking of ZnTCPP molecules immobilized on the TiO_2_ surface, rather than to the formation of a separate crystalline porphyrin phase. Similar low-angle reflections have been reported for ZnTCPP-based assemblies, where a (004) reflection at 17.8° corresponding to a *d*-spacing of approximately 0.49 nm was assigned to ordered porphyrin stacking motifs.^46^ The appearance of these low-angle diffraction features, combined with the retention of the anatase diffraction pattern, provides strong evidence for successful surface sensitization of TiO_2_ with ZnTCPP, while maintaining the crystallinity required for photocatalytic performance is maintained. These findings are consistent with previous studies on porphyrin-TiO_2_ hybrid systems prepared *via* adsorption-based sensitization strategies.^45,47^

#### 3.1.5 SEM of TiO_2_ and TiO_2_-MP

**Fig. 4:**
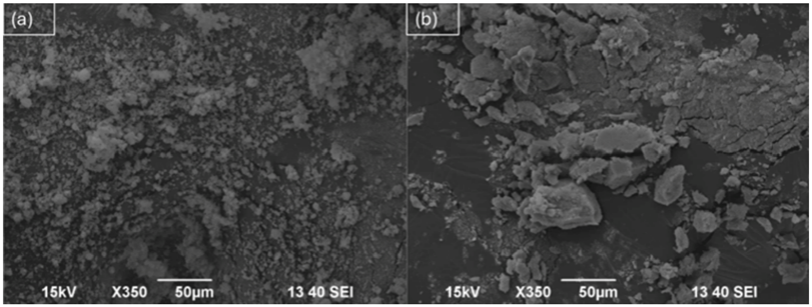
SEM image of (a) pure TiO_2_ and (b) TiO_2_-ZnTCPP.

Scanning electron microscopy (SEM) analysis further supported the successful surface modification of TiO_2_ by ZnTCPP (Fig. 4). Pristine TiO_2_ nanoparticles (Fig. 4a) exhibited the typical angular morphology associated with commercial powders, characterized by well-defined particle edges and relatively smooth surface features. In contrast, the TiO_2_-ZnTCPP hybrid material (Fig. 4b) showed a noticeable change in surface texture. The particle surfaces appeared roughened, with less sharply defined edges and the presence of a thin coating layer. This morphological modification is consistent with the deposition of an organic porphyrin layer on the TiO_2_ surface, most mediated through coordination interactions between the carboxylate groups of ZnTCPP and surface Ti sites. Despite these surface changes, the overall particle shape and size distribution remained largely unchanged. This observation indicates that the sensitization process resulted in surface functionalization rather than bulk structural alteration or particle aggregation. Such behavior is desirable, as it indicates that the sensitization process preserves the overall particle morphology, while introducing a visible-light-responsive porphyrin layer.^48,49^ The combination of unchanged anatase PXRD patterns (Section 3.1.3, Fig. 3) and SEM imaging (Fig. 4) suggests that the textural characteristics of the TiO_2_ support are largely maintained after ZnTCPP immobilization, in agreement with previous reports on porphyrin-sensitized TiO_2_ systems.^50–52^

The formation of a porphyrin-derived surface layer is expected to enhance light harvesting in the visible region without compromising the intrinsic properties of the semiconductor support. These morphological observations are in good agreement with previously reported porphyrin-sensitized TiO_2_ systems prepared using similar surface adsorption approaches.^45,47,53^

#### 3.1.6 Photocatalytic performance

Overall, the combined spectroscopic and structural data support a coherent picture of the TiO_2_-ZnTCPP hybrid. UV-Vis and FT-IR spectroscopy confirm successful metalation and ester hydrolysis, yielding a ZnTCPP species with red-shifted Q bands and surface-anchoring carboxylate groups, which are essential for visible-light harvesting and robust immobilization. PXRD shows that the anatase lattice of TiO_2_ is preserved upon sensitization, while the appearance of low-angle reflections indicate partially ordered stacking of ZnTCPP on the TiO_2_ surface rather than formation of a separate porphyrin phase. SEM images reveal roughened particle surfaces and a thin organic coating, consistent with a porphyrin layer conformally covering the TiO_2_ nanoparticles without inducing aggregation. In conjunction with the radical scavenger experiments and the proposed dye-sensitized mechanism, these results indicate that ZnTCPP forms a strongly coupled interfacial layer that extends the photoresponse of TiO_2_ into the visible region and facilitates interfacial charge transfer, thereby underpinning the enhanced BPA degradation observed under visible-light irradiation.

### 3.2 Photocatalytic degradation of BPA with the hybrid porphyrin-TiO_2_ material

#### 3.2.1 Preliminary hydrolysis and photolysis experiments

Hydrolysis experiments were conducted to assess the structural stability of BPA under prolonged aqueous exposure. A BPA solution (20 mg L^−1^, 10 mL) was magnetically stirred in the dark at ambient temperature and atmospheric pressure to eliminate any contribution from photochemical processes. Aliquots were collected at predetermined time intervals (2, 7, 14, 21, 60, and 90 d) to monitor potential hydrolytic degradation. The experimental results indicated that the compound remained essentially unchanged throughout the entire monitoring period, indicating high hydrolytic stability (Fig. S13). This behaviour was expected, since BPA does not contain chemically labile functional groups that are susceptible to nucleophilic attack by water molecules. Although BPA contains two covalently bound hydroxyl groups, these functionalities do not promote hydrolysis under the examined conditions, as confirmed by the absence of detectable structural degradation.

Photolysis experiments were performed to evaluate the photochemical stability of BPA and the potential contribution of light irradiation to compound degradation. The behaviour of BPA under direct photolysis was compared with that under photocatalytic conditions to isolate the photocatalyst's specific role and determine whether it is necessary for effective degradation. For this purpose, four BPA aqueous solutions (20 mg L^−1^, 10 mL each) were subjected to visible-light irradiation at different pH values (3, 5, 7, and 9) to evaluate the influence of acidity and alkalinity on light-induced degradation.

The experimental results demonstrated minimal to negligible degradation of BPA under photolysis across all the tested solutions (Fig. S14). Slight degradation was observed under acidic conditions, whereas no reduction in absorption was detected at neutral or alkaline pH. At pH 3, the maximum degradation reached approximately 5.0%, whereas at pH 5, it was approximately 6.0% after 180 min of irradiation. These low degradation percentages confirm the stability of BPA under visible-light conditions. At a neutral pH, no photodegradation was observed; however, a slight increase in the solution's absorbance over time was observed, attributed to elevated laboratory temperature on the day of the experiment due to external environmental factors. Under alkaline conditions, BPA exhibited complete photochemical stability, showing no detectable degradation throughout the irradiation period.

### 3.3 Effect of operational parameters

#### 3.3.1 Effect of porphyrin loading on the photocatalytic performance of TiO_2_

**Fig. 5:**
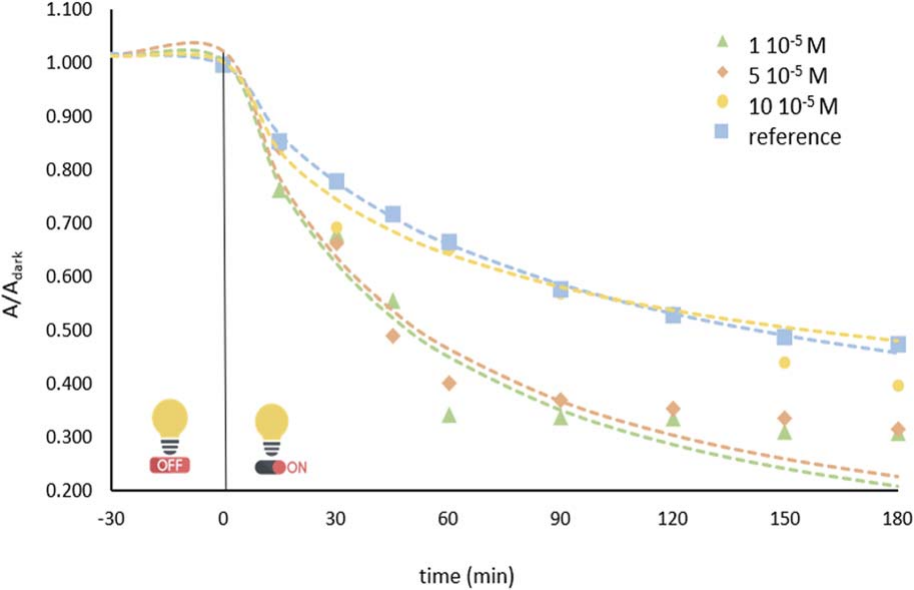
Effect of porphyrin loading in the hybrid material on BPA degradation: [catalyst] = 0.5 mg L^−1^, [BPA]_0_ = 20 mg L^−1^.

Porphyrin loading constitutes a critical factor, as it governs the efficiency of light absorption, charge transfer, and the subsequent generation of ROS. Experiments were conducted by varying the porphyrin load in TiO_2_-based photocatalytic systems with incremental amounts of porphyrin corresponding to 1 × 10^−5^, 5 × 10^−5^, and 1 × 10^−4^ M, with 20 mg L^−1^ BPA reaction solution at pH 7; the results are shown in Fig. 5. Within 180 min of irradiation, the composites containing 1 × 10^−5^ and 5 × 10^−5^ M porphyrin exhibited comparable BPA degradation efficiencies. The system with the highest porphyrin loading (1 × 10^−4^ M) exhibited significantly lower activity, which was attributed to the active sites becoming saturated as extensive porphyrin coverage of the TiO_2_ surface.

The porphyrin layer may function as a light barrier, reducing photoexcitation of TiO_2_ by limiting photon penetration. Hence, the 1 × 10^−5^ M porphyrin-TiO_2_ composite was determined to be the optimal formulation because it exhibited the highest BPA removal, while requiring a lower sensitizer content.

#### 3.3.2 Effect of initial solution pH

**Fig. 6:**
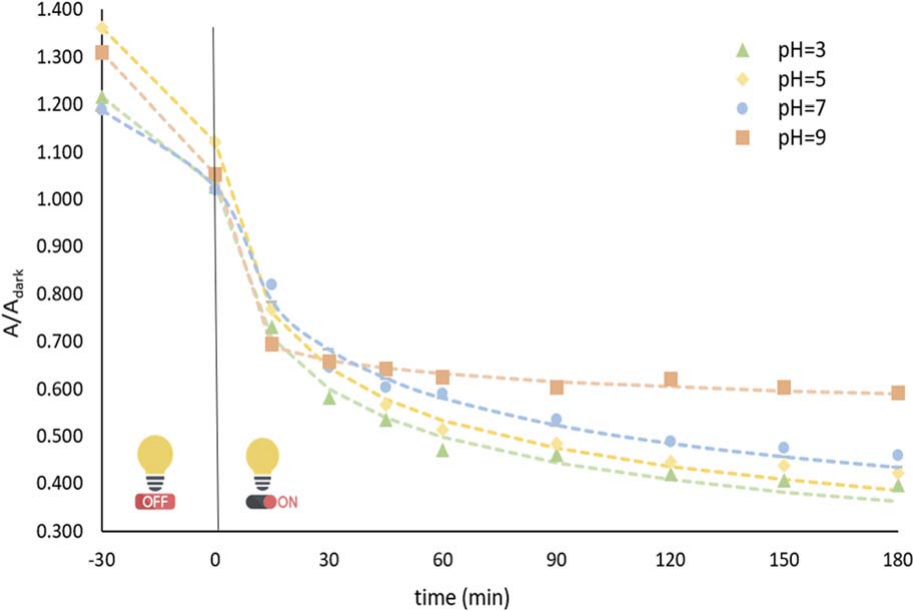
Effect of pH on BPA degradation: [catalyst] = 0.5 mg L^−1^, [BPA]_0_ = 20 mg L^−1^.

pH is a critical parameter that significantly influences the photocatalytic process by affecting the photocatalyst's surface charge.^54^ The effect of solution pH on BPA degradation was investigated in the pH range of 3–9. The experimental results demonstrated that the degradation efficiency increased when the solution was switched from alkaline to acidic conditions. As shown in Fig. 6 the highest BPA removal was achieved at a pH of 3, highlighting the favourable role of acidic conditions in enhancing photocatalytic activity.

#### 3.3.3 Effect of initial BPA concentration

**Fig. 7:**
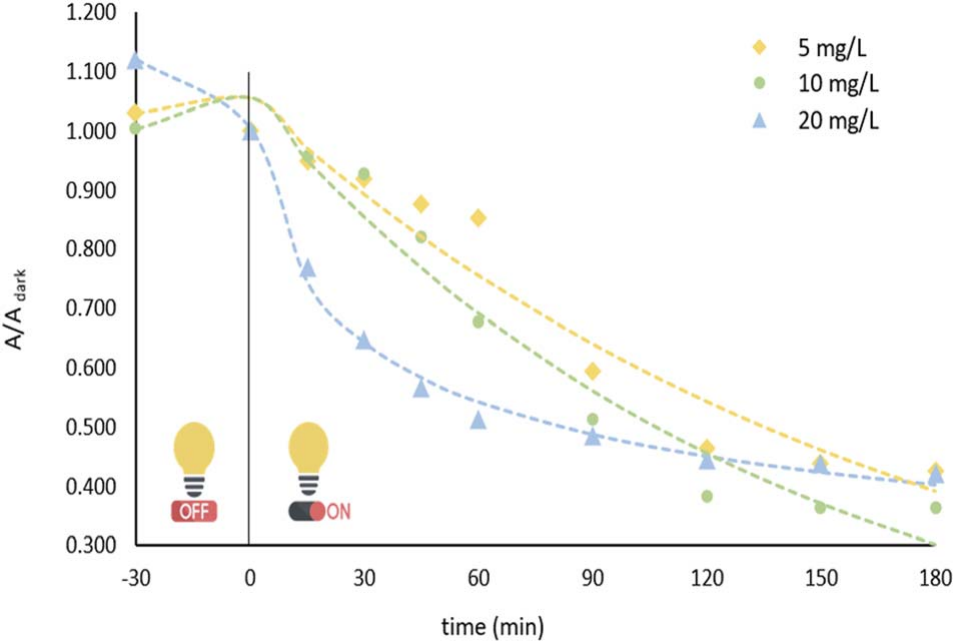
Effect of the initial BPA concentration over power law kinetics: pH = 3, [catalyst] = 0.5 mg L^−1^, [BPA]_0_ = 5, 10, and 20 mg L^−1^.

The negative impact of high pollutant concentrations on the photocatalytic process has been highlighted in numerous studies.^55^ Because the initial concentration of BPA can influence the active sites of the photocatalyst, leading to variations in the degradation rate, photocatalytic experiments were performed using BPA solutions with concentrations of 5, 10, and 20 mg L^−1^. The data obtained indicate that higher substrate concentrations yield lower *k* values. The highest removal rate was observed at 10 mg L^−1^, where a degradation efficiency of 63.87%, as shown in Fig. 7.

The increase in pollutant concentration leads to saturation of the active sites of the photocatalysts, resulting in lower formation of ROS as the interaction between the photocatalyst and water molecules becomes less efficient. This explains the superior performance observed at 10 mg L^−1^ relative to 20 mg L^−1^. At 5 mg L^−1^, we assumed that BPA was highly dispersed in the solution, thereby limiting effective contact with the photocatalyst surface and resulting in a lower degradation rate.

#### 3.3.4 Effect of H_2_O_2_ addition on photocatalytic performance

**Fig. 8:**
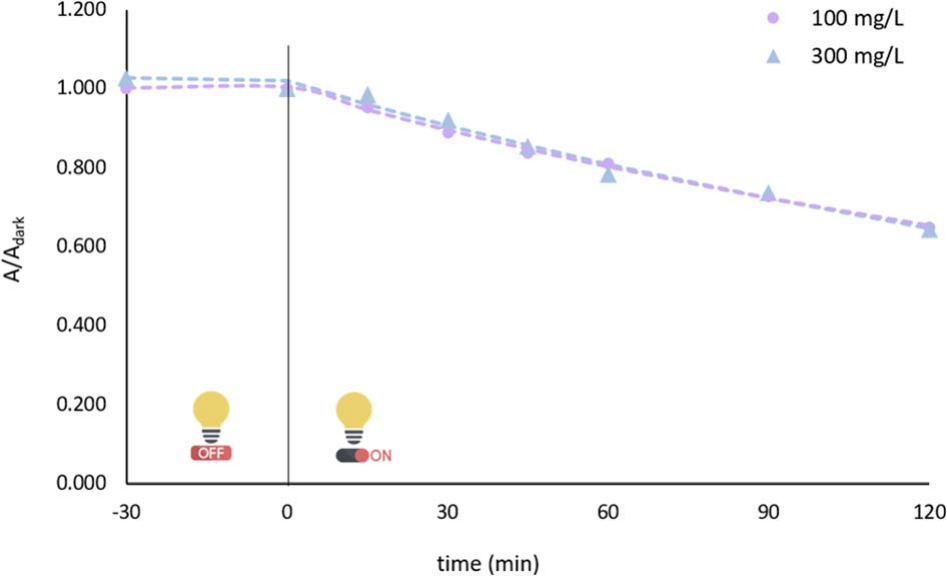
Effect of H_2_O_2_ addition on photocatalytic performance, pH = 3, [catalyst] = 0.5 mg L^−1^ [BPA]_0_ = 20 mg L^−1^.

It is well established that in light-driven oxidation processes, including direct photolysis and heterogeneous photocatalysis, the addition of oxidizing agents promotes ROS formation *via* photochemical activation under light irradiation.^56,57^ In the present study, hydrogen peroxide (H_2_O_2_) was introduced as an external oxidant at concentrations of 100 and 300 mg L^−1^ to evaluate its effect on BPA degradation. The results illustrated in Fig. 8 indicate that the addition of the oxidant led to lower removal efficiencies (35.06% and 37.55%, respectively) compared with the blank system, suggesting an inhibitory effect.

The influence of H_2_O_2_ in photocatalytic systems is known to be multifaceted.^58^ On the one hand, H_2_O_2_ can undergo photolytic activation to generate hydroxyl radicals according to reaction (1), thereby potentially enhancing pollutant degradation.^59^ On the other hand, H_2_O_2_ may partially absorb incident light, reducing the photon flux available for catalyst excitation and ROS formation. In addition, H_2_O_2_ can act as a scavenger of reactive oxygen species through the reaction pathways shown in reactions (2)–(5),^59^ leading to a decrease in the steady-state concentration of highly reactive radicals. Based on the observed degradation trends, the scavenging pathways appear to dominate under the present experimental conditions, resulting in suppression rather than enhancement of BPA removal.

1 H_2_O_2_ + *hν* → 2HO^˙^

2 H_2_O_2_ + HO^˙^ → HO_2_^˙^ + H_2_O

3 H_2_O_2_ + HO_2_^˙^ → HO^˙^ + O_2_ + H_2_O

4 2HO_2_^˙^ → H_2_O_2_ + O_2_

5 HO^˙^ + 2HO_2_^˙^ → O_2_ + H_2_O

Notably, previous studies have also reported that excessive H_2_O_2_ concentrations can adversely affect photocatalytic degradation efficiency due to radical recombination and scavenging effects, in agreement with the behavior observed in the present work.^60,61^

#### 3.3.5 Effect of radical scavengers

To clarify the role of the main reactive species involved in the photocatalytic degradation of BPA, quenching experiments were performed using selective radical scavengers, and the reaction products were analyzed using LC-Orbitrap HRMS. Isopropanol (IPA), *p*-benzoquinone (*p*-BQ), and EDTA were employed as scavengers for hydroxyl radicals (HO^˙^), superoxide species (O_2_^˙−^), and photogenerated holes (h^+^), respectively. The concentration of each scavenger was kept constant at 0.5 mol L^−1^ throughout the experiments. The addition of *p*-BQ resulted in a pronounced suppression of BPA degradation, indicating that O_2_^˙−^ plays a dominant role in driving the photocatalytic reaction. Upon the addition of IPA, a noticeable decrease in degradation efficiency was also observed. Although this inhibition was slightly less pronounced than that caused by *p*-BQ, it nevertheless confirms that hydroxyl radicals (OH^˙^) contribute significantly to the oxidation process. In contrast, the introduction of EDTA caused only a minor reduction in BPA degradation, indicating that the photogenerated holes (h^+^) played a comparatively less significant role in the overall photocatalytic mechanism. Overall, these results demonstrate that BPA degradation is primarily governed by radical-mediated oxidation, with the relative contribution of the reactive species following the order: O_2_^˙−^ ≈ HO > h^+^.

### 3.4 Kinetic study

The kinetic behavior of the system was described using a power-law type model, consistent with non-classical relaxation dynamics frequently reported in complex and heterogeneous media. The temporal evolution of the response variable was fitted using the following expression:
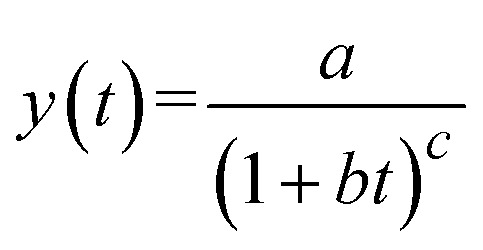
where a represents the initial magnitude of the observable, *b* is a characteristic rate parameter (with *b* = *k*), and *c* is a scaling exponent governing the temporal decay.^62^

This analytical solution corresponds to a power-law kinetics, a framework dissimilar to classical integer-order exponential models, characterized only by time scale. In contrast, the current formulation essentially embraces a distributed time scale and memory effects, features typically associated with fractal-like or fractional kinetic behavior.^63^ The exponent *c* provides a measure for the deviation from classical first-order kinetics: *c* = 1 corresponds to simple hyperbolic decay, whereas non-integer values of “*c*” represent irregular transport or relaxation processes as consequence of structural heterogeneity and spatial limitations.^62,64^

Such power-law forms have been widely used to describe adsorption kinetics, anomalous diffusion, and relaxation processes in complicated systems, where the dynamics cannot be sufficiently described by conventional integer-order rate equations. In this context, “*k*” (embedded in *b*) is an effective kinetic constant, while the “*c*” accounts the degree of kinetic heterogeneity and possible fractals in the system.^65^

Hence, the proposed model offers a physically interpretable and mathematically versatile framework to describe non-exponential kinetic phenomena, bridging classical rate theory with generalized fractal or fractional kinetic models.

The concentration values were determined spectroscopically and fitting was performed using Gnuplot to extract the rate constants and correlation coefficients. Kinetic comparisons were made between the photolysis, hydrolysis, and photocatalytic treatments as well as across different pH values, BPA/catalyst loadings, and irradiance intensities.

The overall kinetic parameters obtained by fitting the power-law decay model are summarized in Table 1. The factors include the BPA's solution initial absorbance (*A*_0_), the rate constant (*k*), the half-life (*t*_1/2_), the exponent (*n*) and the coefficient of determination (*R*^2^), for all the experimental conditions investigated in this study.

**Table 1:** Kinetic parameters of the power-law decay model under different experimental conditions

Experimental conditions		Kinetic parameters				
		*A*_0_	*k* (min^−1^)	*n*	*R* ^2^	*t*_1/2_ (min)
Porphyrin load (M)	1 × 10^−5^	1.038	0.0399	0.629	0.9620	50.28
	5 × 10^−5^	1.024	0.0384	0.661	0.9524	48.27
	10 × 10^−5^	1.018	0.04566	0.326	0.9790	161.2
pH	3	1.122	0.2196	0.275	0.9955	51.96
	5	1.030	0.2055	0.275	0.9910	55.70
	7	1.025	0.1218	0.265	0.9870	104.0
	9	1.053	73.06	0.0607	0.9970	1235.7
[BPA] (mg L^−1^)	5	1.066	5.28 10^−6^	1069.2	0.9500	122.7
	10	1.064	5.89 10^−6^	1179.3	0.9560	99.85
	20	1.122	0.220	0.2752	0.9955	51.98

For the porphyrin load, while 1 × 10^−5^ and 5 × 10^−5^ M indicated comparable results (50.28 and 48.27 min half-life values, respectively), the lower porphyrin concentration was chosen for further experimental tests due to practical considerations. Regarding pH, the optimal kinetic profile was observed in pH 3 and was accordingly chosen. Lastly, the initial BPA concentration on 20 mg L^−1^ revealed the lowest half-life and was subsequently used throughout this work.

### 3.5 Reusability and stability

The reusability of the synthesized Porphyrin-TiO_2_ catalyst was evaluated by testing it in three consecutive cycles. As inferred from Table S3, the rate constants were similar between the first two cycles, indicating that the stability of the catalyst was sufficient. However, after the second cycle, the catalyst lost some of its ability.

### 3.6 Comparative evaluation with reported metalloporphyrin catalysts

To place the performance of the TiO_2_-ZnTCPP hybrid in context, we compared it with representative metalloporphyrin-based systems reported for the degradation of BPA and structurally related phenolic pollutants (Table 2). These include homogeneous Fe(iii)-meso-tetra(4-carboxyphenyl)porphyrin (Fe-TCPP) Fenton-like catalysts activated by H_2_O_2_,^66^ ferrate(VI) systems mediated by protoporphyrin IX,^67^ heterogeneous Sn(iv)-5,10,15,20-tetraphenylporphyrin immobilized on silica,^68^ porphyrinic Zr-MOFs such as PCN-223,^69^ and a Co-5,10,15,20-tetra(4-pyridyl)porphyrin (Co-TpYp) MOF coupled with TiO_2_ for PMS activation.^70^ While Fe-TCPP/H_2_O_2_ and ferrate(VI)-PpIX systems can achieve very rapid BPA removal, they operate in homogeneous phase and require continuous dosing of strong oxidants, which complicates catalyst recovery and may generate secondary sludge or residual oxidants. In contrast, heterogeneous SnP-silica and PCN-223 exploit ^1^O_2_ and O_2_˙^−^ as the main oxidants and offer good reusability, but either show self-sensitized porphyrin degradation upon cycling (SnP-silica) or rely on more complex MOF architectures and, in some cases, high salinity to reach their best performance.

**Table 2:** Comparison of metalloporphyrin-based catalytic systems for BPA and phenolic-related pollutants

Catalyst system	Porphyrin used	Light source	Target pollutant	Degradation %	Time (min)	Rate constant *k* (min^−1^)	Catalyst (g L^−1^)	Dominant ROS	pH range/optimum	Reusability	Ref.
Fe-TCPP/H_2_O_2_	Fe(iii)-meso-tetra(4-carboxyphenyl)porphyrin (TCPFe)	None (dark Fenton-like)	BPA, phenol, 4-CP, TCP, 2-CP	>97% BPA in 0.5 min (pH 12); >60% over pH 4–12	0.5–15	∼0.04–>1.0 (non-linear; >0.04 at pH 4–7)	Fe 0.02 mM; H_2_O_2_4 mM	por-Fe(iv)=O, por-Fe(iv)-OH >> ˙OH, O_2_˙^−^, H˙	4–12 (fastest alkaline)	Homogeneous; 5 cycles with 4-CP (some loss)	66
Ferrate(VI)-PpIX mediator system	Protoporphyrin IX (free base) with Fe(vi)	None (chemical oxidation)	Phenolic pollutants incl. BPA	Near-complete in minutes at µM–mM Fe(vi)	Few min	Up to ∼0.1–0.3 (substrate-dependent)	µM PpIX; sub-mM Fe(vi)	High-valent Fe(v)/Fe(iv), ^1^O_2_, ˙OH	Near neutral-slightly alkaline	Homogeneous; no solid catalyst to recover	67
SnP-silica	Sn(iv)-5,10,15,20-tetraphenylporphyrin (Sn(OH)_2_TPP) on silica	Fluorescent, UVA, Xe; visible including *λ* > 400 nm	Pharmaceuticals; phenolics incl. BPA	Strong for phenolates; >80% for many phenols in 120 min (alkaline)	120	FFA: 2.358 h^−1^ (∼0.0393 min^−1^); drugs 0.33–7.53 h^−1^	0.5 g L^−1^ SnP-silica	^1^O_2_ dominant; at high power, direct 3SnP* ET to substrates	Neutral to alkaline; phenol/BPA faster at high pH	Activity gradually decreases over 5 cycles	68
PCN-223 (Zr-TCPP)	H_2_TCPP in Zr-based MOF PCN-223	500 W Xe, *λ* > 420 nm	Bisphenol F (15 mg L^−1^)	∼98% in 120 min (0.2 g L ^−1^)	120	0.020–0.035; up to 0.0443 with 500 mM SO_4_^2-^	0.2 g L^−1^	^1^O_2_ + O_2_˙^−^ >> ˙OH (salt-assisted ˙OH at high salinity)	pH 3–9; highly salt-tolerant	>78% after 8 cycles (±500 mM NaCl)	69
CTT20 (Co-TpYp/TiO_2_)+PMS	Co(ii)-5,10,15,20-tetra(4-pyridyl)porphyrin in MOF (Co-TpYp)	50 W visible LED, *λ* > 420 nm	BPA (20 mg L^−1^)	94.1% (Vis/CTT20); ∼100% (Vis/CTT20/PMS, 60 min)	60	0.0444 (Vis/CTT20); 0.0730v(Vis/CTT20/PMS)	0.4 g L^−1^ PMS 1.2 mM	SO_4_ − + ˙OH + ^1^O_2_ (PMS-activated) > O_2_˙^−^	Effective pH 3–9; tests at pH 7	Good stability; repeated runs (no major loss)	70
CuTCPP/TiO_2_	meso-tetra(4-carboxyphenyl)porphyrinato copper(ii)	Fluorescent lamp (30 W, 5.02 mW cm^−2^ irradiance)	Methylene blue (10 mL, 15.6 µM)	99%	180	—	2 µM	O_2_˙^−^	Effective pH 1	No change in photo catalaytic activity with different solvents	71
TiO_2_-ZnTCPP hybrid	Zn(ii)-meso-tetra(4-carboxyphenyl)porphyrin (ZnTCPP) adsorbed on TiO_2_	100 W visible white LED, *λ* > 420 nm	BPA (5–20 mg L^−1^)	Up to 63.9% at 10 mg L^−1^, pH 3 (180 min); lower at 20 mg L^−1^	180	Power-law effective *k* > bare TiO_2_/photolysis (insert numeric value)	0.5 g L^−1^; optimal porphyrin loading ∼1 × 10^−5^ M	O_2_^−^ > ˙OH >> h+ (dye-sensitized electron injection)	Best at pH 3; decreasing to neutral/alkaline	Solid photocatalyst; similar *k* for 2 cycles, moderate loss by 3rd	This work

The Co-TpYp/TiO_2_ hybrid exemplifies a different strategy, where a porphyrin-based MOF acts as a visible-light antenna and PMS activator, yielding synergistic photocatalysis-sulfate-radical oxidation with high apparent rate constants for BPA. However, this approach intrinsically depends on peroxymonosulfate dosage and on a core–shell MOF/TiO_2_ structure. By comparison, the TiO_2_-ZnTCPP photocatalyst developed in this work is constructed through a straightforward adsorption-based sensitization of commercially available anatase TiO_2_ with ZnTCPP, without forming a separate MOF phase or using external oxidants. Under visible-light irradiation, BPA degradation proceeds *via* a dye-sensitized route in which photoexcited ZnTCPP injects electrons into the TiO_2_ conduction band, leading to sequential formation of O_2_˙^−^, H_2_O_2_, and –OH, with O_2_˙^−^ and –OH identified as the principal reactive oxygen species, and photogenerated holes playing a secondary role. Although the absolute degradation rate and optimum pH (acidic conditions) differ from those of some benchmark systems, our hybrid offers a distinct combination of features: a simple and scalable synthetic route, operation solely under visible light and dissolved oxygen, absence of added oxidants (H_2_O_2_ or PMS), solid-phase catalyst recoverability, and a detailed power-law kinetic and toxicity assessment of transformation products. Collectively, these characteristics differentiate TiO_2_-ZnTCPP from previously reported metalloporphyrin systems and highlight its potential as a robust and conceptually complementary platform for BPA remediation.

### 3.7 Identification of TPs

Because TPs can be more recalcitrant or even more toxic than the parent compound itself, identifying the emerging TPs was considered essential for an integrated assessment of the applied treatment. The main TPs were identified based on the information generated from LC-HRMS analysis. Molecular formulas could be proposed based on the masses of the pseudo-molecular ion peaks that were detected with an accuracy of four decimals, and the distinctive fragments obtained from MS/MS offered additional insight into their potential structures. Most of these TPs eluted earlier than the parent compound, suggesting that they were more polar. The MS2 fragmentation of the precursor BPA yielded five fragments, with *m*/*z* 211.0759 (a and b), 244.0736, 133.0653, and 93.0334 (Table 3), which were considered during the TPs elucidation for the assignment of the possible structures of the detected TPs.

**Table 3:** LC-HRMS data of the parent compound and its major TPs

Compound name	*t*_R_ (min)	[M *−* H]^−^	Theoretical mass (*m*/*z*)	Experimental mass (*m*/*z*)	Mass accuracy (Δ)	Fragments (*m*/*z*)
BPA	7.50	C_15_H_15_O_2_^−^	227.2795	227.2789	−2.639	244.0736
						211.0759
						133.0653
						93.0334
TP242	7.71	C_15_H_13_O_3_^−^	241.2630	241.2632	0.8289	
TP134	3.20	C_9_H_9_O^−^	133.1680	133.1678	−1.5019	133.0653
						93.0334
TP150	2.63	C_9_H_9_O_2_^−^	149.1674	149.1688	9.3854	93.0334
TP136a	3.68	C_9_H_11_O^−^	135.1839	135.1845	4.4384	133.0653
						93.0334
TP136b	3.48	C_8_H_7_O_2_^−^	135.1408	135.1411	2.2199	133.0653
						93.0334
TP200	11.80	C_13_H_11_O_2_^−^	199.2263	199.2268	2.5097	133.0653
TP214	9.01	C_14_H_13_O_2_^−^	213.2529	213.2531	0.9379	211.0759
TP244	5.90	C_15_H_15_O_3_^−^	243.2789	243.2793	1.6442	211.0759

**Fig. 9:**
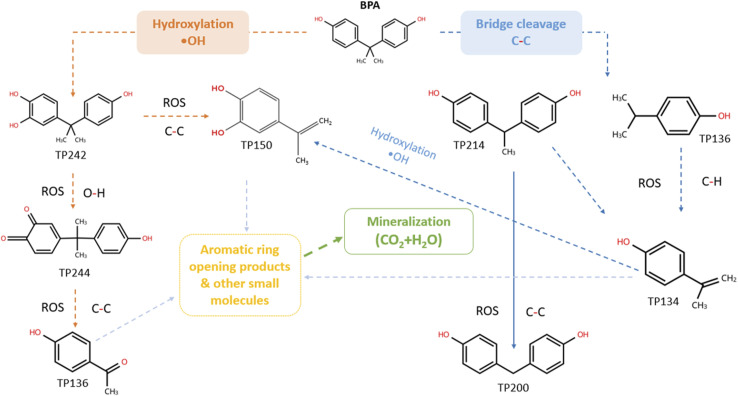
Proposed pathway for the photocatalytic degradation of BPA, governed by two main routes (i) hydroxylation of the aromatic rings, and (ii) cleavage of the central C–C bridge.

As illustrated in the proposed transformation mechanism (Fig. 9), two main degradation pathways dominate BPA photocatalysis: (i) hydroxylation of the aromatic rings and (ii) cleavage of the central C–C bridge of BPA. Both pathways ultimately result in the breakdown and mineralization of the parent compound.

In the first pathway, OH radicals attack the parent molecule, resulting in a mono-hydroxylated derivative (TP244). Subsequently, reactive oxygen species (ROS) oxidize the benzylic group to a carbonyl group, yielding TP242. Further ROS-induced C–C bond cleavage of the side chain resulted in a smaller TP136a.

In the second pathway, it is obvious that the cleavage of the central C–C bridge of the parent compound is favoured by ROS, generating TP136b. The latter undergoes C–H abstraction, which leads to TP134. Further oxidation of TP214, specifically through C–C bond cleavage, produces TP200. On the other hand, TP134 can be further hydroxylated by ˙OH, forming di-OH TP150. All TPs and intermediates stemming from both pathways may be further attacked by ROS (hydroxylation, dehydrogenation, C–C bond scission) and lead to aromatic ring-opening products of lower molecular weights before final mineralization to CO_2_ and H_2_O.

## 4 Proposed mechanism

**Fig. 10:**
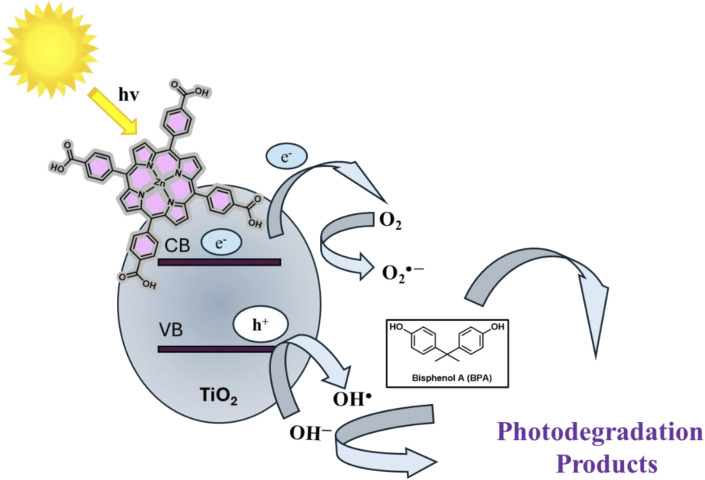
Proposed photocatalytic degradation mechanism of BPA with TiO_2_-MP.

The photocatalytic degradation of bisphenol A (BPA) over the TiO_2_-MP hybrid under visible-light irradiation is proposed to proceed *via* a dye-sensitized mechanism that synergistically couples the strong light-harvesting capability of the metalloporphyrin photosensitizer with the redox activity of TiO_2_ and dissolved molecular oxygen (Fig. 10).^72^ Upon visible-light absorption in the Soret or Q bands, the metalloporphyrin (MP) is excited from its ground state to the singlet excited state (^1^MP*), followed by efficient intersystem crossing to a long-lived triplet excited state (^3^MP*). Because of the favourable energetic alignment between the excited-state redox potential of the metalloporphyrin and the conduction band (CB) edge of TiO_2_, the triplet state can inject an electron into the TiO_2_ CB.^26,73,74^ This interfacial electron transfer generates a charge-separated state consisting of an electron in the TiO_2_ CB and an oxidized porphyrin radical cation (MP^˙+^).

The injected electrons migrate through the TiO_2_ lattice and reduce the dissolved oxygen molecules to superoxide radicals (O_2_˙^−^). These reactive species subsequently undergo protonation and disproportionation reactions, leading to the formation of hydrogen peroxide (H_2_O_2_) and additional hydroxyl radicals (^˙^OH). In parallel, the oxidized metalloporphyrin is regenerated to its ground state through electron donation, either directly from BPA molecules or from intermediate reductive species formed during the degradation process.

Simultaneously, photogenerated holes remaining in the valence band (VB) of TiO_2,_ as well as high-valent porphyrin radical cations can oxidize surface-adsorbed water molecules or hydroxide ions to produce additional hydroxyl radicals (^˙^OH). The combined action of superoxide, hydroxyl radicals, and other reactive oxygen species (ROS) creates a highly oxidative environment that attacks BPA molecules through successive hydroxylation, aromatic ring opening, and oxidative fragmentation reactions, ultimately leading to mineralization into CO_2_ and H_2_O (Fig. 10). The strong electronic coupling between the metalloporphyrin and TiO_2_, facilitated by carboxylate anchoring and intimate interfacial contact, promotes efficient spatial separation of photogenerated charge carriers and suppresses electron–hole recombination. This synergistic interaction is therefore responsible for the enhanced visible-light-driven BPA degradation efficiency observed for the TiO_2_-metalloporphyrin hybrid compared with bare TiO_2_ under identical experimental conditions.^15,74–76^

The superior performance of TiO_2_-ZnTCPP compared to bare TiO_2_ under visible light (Section 3.3), combined with ROS scavenging results identifying –O_2_^−^ and –OH as dominant species (Section 3.3.5), provides strong indirect evidence for this sensitization mechanism. The observed enhancement in visible-light activity and ROS generation aligns with established porphyrin/TiO_2_ interfacial electron transfer processes reported in the literature.^71,77^

## 5 *In silico* toxicity assessment (ECOSAR)

**Fig. 11:**
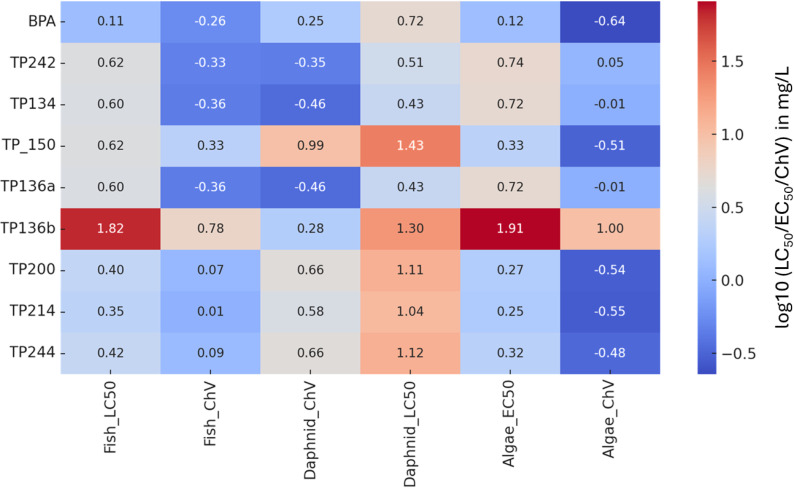
Heatmap illustrating ECOSAR results for the predicted acute/chronic toxicity of the TPs (LC50, half lethal concentration; EC50, half effective concentration; ChV, chronic value).

Transformation products are generally expected to be less toxic than their precursors; sometimes, hazardous or highly toxic compounds may arise from transformation pathways.^78^ To this end, ECOSAR was used to perform *in silico* toxicity assessments focusing on acute and chronic endpoints, to analyze the potential hazardous effects of BPA and its resulting TPs. Table S4 shows the anticipated values of the endpoints, as well as for log *K*_ow_ and water solubility, while Fig. 11 illustrates ECOSAR results for the predicted acute/chronic toxicity of the TPs.

BPA itself exhibited high toxicity across all aquatic species tested, with predicted LC50 values at 1.28 mg L^−1^ and chronic value (ChV) at 0.55 mg L^−1^ for fish, placing it in the category of “toxic to aquatic life” according to EU and EPA regulations.^6^ Following the photocatalytic degradation, the major TPs exhibited markedly reduced toxicity. ECOSAR modelling results showed acute (LC50, EC50) and chronic (ChV) values for the TPs substantially higher than those of BPA, ranging from 2.24 to 66.5 mg L^−1^ (fish LC50) and 0.28 to 9.95 mg L^−1^ (ChV), respectively. In general, hydroxylation-based pathways (TP244, TP242, TP150, TP136b) generate intermediates that largely preserve the biphenyl backbone of BPA. The most abundant intermediates, TP136b and TP150, presented very low risk levels, with LC50 values exceeding 66 mg L^−1^, which are well above the standard regulatory safety thresholds for wastewater and environmental discharge. These products retain phenolic groups capable of redox cycling and endocrine-receptor binding, which explains their relatively high predicted toxicity, particularly TP136b. On the other hand, bridge-cleavage pathways (TP134, TP200, TP214) produce smaller, less hydrophobic fragments with reduced structural similarity to BPA. These products show substantially lower predicted toxicity across all three target organisms, consistent with their advanced oxidation state and diminished biological activity. Transformation products dominated by BPA-like fragments (*m*/*z* 211–244) correlate with higher toxicity, whereas products characterized by simple phenolic fragments (*m*/*z* 93–133) correlate with detoxification.

A comparison of log *K*_ow_ and water solubility values indicated greater aqueous solubility and substantially lower bioaccumulation potential for TPs *versus* BPA, suggesting a further reduction in ecological risk. These data support the conclusion that photocatalytic treatment not only efficiently removes BPA but also yields degradation products with minimal toxicological impact on aquatic organisms. Overall, the toxicity profile of the system shifts from “high risk” in the presence of BPA to “very low risk” or “non-toxic” after photocatalytic transformation, confirming the environmental safety and effectiveness of the hybrid TiO_2_-metalloporphyrin catalyst for BPA remediation.

## 6 Conclusions

This study demonstrates that surface sensitization of TiO_2_ with ZnTCPP represents an effective strategy for developing visible-light-active photocatalysts for aqueous contaminant remediation. The hybrid material achieves efficient BPA degradation through a radical-mediated mechanism dominated by O_2_^−^ and ^˙^OH species, with HRMS analysis confirming oxidative transformation pathways leading to less toxic intermediates. Power-law kinetic modelling reveals non-classical degradation dynamics influenced by operational parameters, while *in silico* toxicity assessment validates the environmental safety of the treatment process. These findings highlight the potential of porphyrin-TiO_2_ hybrids as sustainable platforms for solar-driven wastewater treatment, offering enhanced photon utilization and reduced ecological risk compared to conventional UV-based TiO_2_ photocatalysis.

## Author contributions

Christina Tsamtzidou: investigation, formal Analysis, visualization, writing – original draft; Athanasios Theodoridis: investigation; Dimitrios-Rafail Bitsos: investigation, writing – original draft; Michail Chalaris: methodology, resources, writing – review & editing; Kalliopi Ladomenou: methodology, resources, writing – original draft, writing – review & editing, supervision; Christina Nannou: methodology, resources, writing – original draft, writing – review & editing, supervision; conceptualization. All authors have read and agreed to the published version of the manuscript.

## Conflicts of interest

The authors have declared that no competing interests exist.

## Supplementary Material

RA-016-D6RA01469K-s001

## Data Availability

All relevant data are within the paper and its supplementary information (SI) files. Supplementary information: a word (docx.) file including four (4) Tables and ten (14) Figures. See DOI: https://doi.org/10.1039/d6ra01469k.
