# Supplementary material for: Metalloporphyrin-based hybrid photocatalyst for bisphenol A degradation: kinetics, HRMS-based analysis of transformation products, and toxicity assessment
Source: RSC Adv. 2026 Apr 13;16(20):18374–88. doi: 10.1039/d6ra01469k (PMC13074201; doi:10.1039/d6ra01469k)
Supplement: RA-016-D6RA01469K-s001 [file RA-016-D6RA01469K-s001.pdf]

Supplementary Information (SI) for RSC Advances.
This journal is © The Royal Society of Chemistry 2026

SUPPLEMENTARY MATERIAL

for

Metalloporphyrin-based hybrid photocatalyst for bisphenol A degradation: kinetics, HRMS-based analysis of transformation products, and toxicity assessment

Christina-Konstantina Tsamtzidou [a], Athanasios Theodoridis [a], Dimitrios Rafail Bitsos [a], Michail Chalaris [a], Kalliopi Ladomenou [a]* and Christina Nannou [a]*

[a]Hephaestus Laboratory, School of Chemistry, Faculty of Sciences, Democritus University of Thrace, GR-65404 Kavala, Greece

Table S1 Gradient elution program

Time (min)	Flow (mL min^{-1})	MeOH + 5mM ammonium formate	H_2O + 5mM ammonium formate
0.0	0.300	10.0	90.0
1.5	0.300	10.0	90.0
4.0	0.300	60.0	40.0
8.0	0.300	70.0	30.0
11.0	0.300	100.0	0.0
13.0	0.300	100.0	0.0
14.0	0.300	10.0	90.0
15.0	0.300	10.0	90.0

Table S2 Operational parameters of LTQ - Orbitrap HRMS instrument

Parameters	Values
Full scan	
Resolution	60,000 FWHM
Mass range	120-400 amu
Mass tolerance	<5 ppm
Spray voltage	3.5 kV
Sheath gas	40 au
Auxiliary gas	10 au
Sweep gas	0 au
Capillary voltage	35 V
Capillary temperature	320°C
Tube lens voltage	90 V
Automatic gain control (AGC) target	1×10^6
Source geometrical position	x*: 2µm, y*: C position, z*: 0
dd MS/MS mode	
Resolution	17,500 FWHM
Normalized collision energy (NCE)	35%
Fragmentation technique	Collision Induced Dissociation (CID)

*x,y,z: *geometrical position of source*; x-*horizontal*, y-*vertical* and z-*diagonal directio*

Table S3 Reusability of the catalyst for three consecutive cycles

	1st run	2nd run	3rd run
**BPA**	69.67	62.08	60.92

Table S4 Acute/chronic toxicity of the TPs, predicted log Kow and chemical classification for BPA TPs as outputs from ECOSAR

Compound Name	MW	ECOSAR Classification	log K_{ow} (est)	Water solubility (est)	Acute toxicity			Chronic toxicity		
					LC50	LC50	EC50	ChV	ChV	ChV
					Fish	Daphnid	Green Algae	Fish	Daphnid	Green Algae
BPA	228.2863	(Poly)Phenols	3.643	85.281	1.28	5.24	1.33	0.55	1.77	0.23
TP214	214.2598	(Poly)Phenols	3.1895	265.01	2.24	11	1.78	1.03	3.82	0.284
TP200	200.2332	Phenols	3.0551	185.44	2.52	13	1.85	1.18	4.56	0.288
TP244	244.2857	(Poly)Phenols	3.1628	192.5	2.65	13.1	2.07	1.22	4.57	0.329
TP134	134.1751	Phenols	2.9621	993.64	3.99	2.67	5.22	0.44	0.35	0.97
TP136a	136.191	Phenols	2.9688	1711.4	4.00	2.69	5.25	0.44	0.35	0.98
TP242	242.0943	Phenols	3.3094	148.02	4.15	3.25	5.51	0.47	0.45	1.11
TP150	150.1745	Phenols	2.4819	2186.6	4.15	26.90	2.16	2.12	9.76	0.31
TP136b	136.1479	Phenols	1.1934	19369	66.50	20.00	81.60	6.07	1.90	9.95

Supplementary Figures

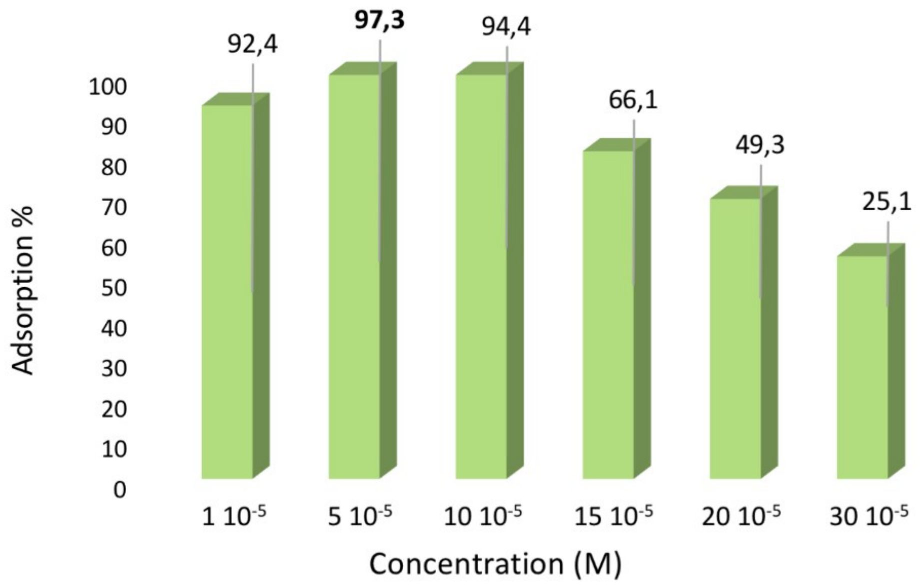

Figure S1 Adsorption efficiencies of the six synthesized TiO_2-ZnTCPP hybrid photocatalyst

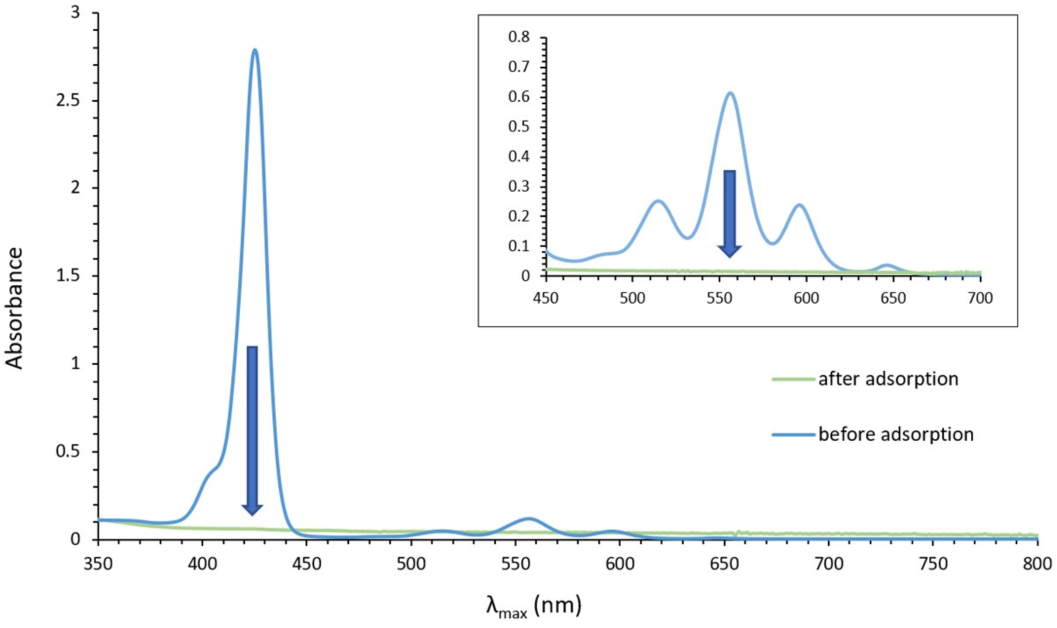

Figure S2 UV-Vis spectra of ZnTCPP (5×10^{-5}) absorption decrease before and after adsorption onto TiO_2

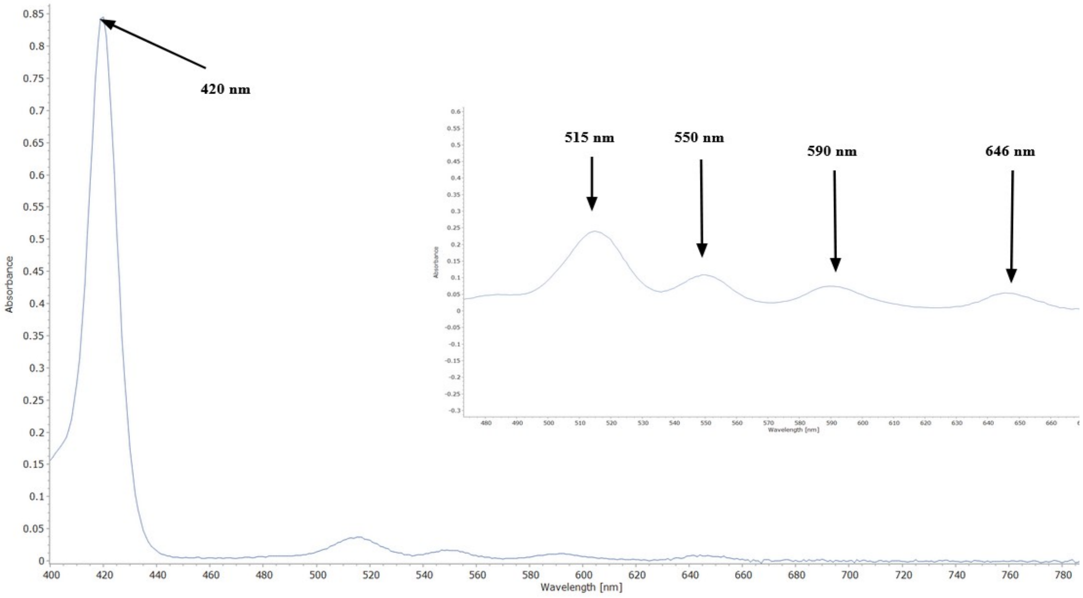

Figure S3 UV-Vis spectra of TPPCOOMe

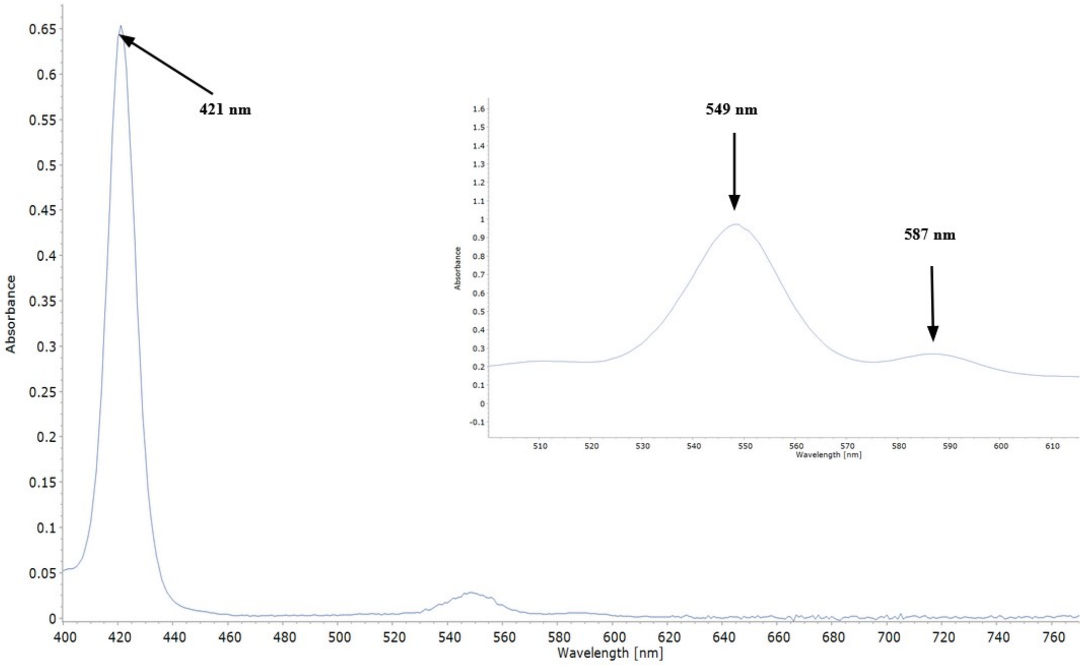

Figure S4 UV-Vis spectra of ZnTPPCOOMe

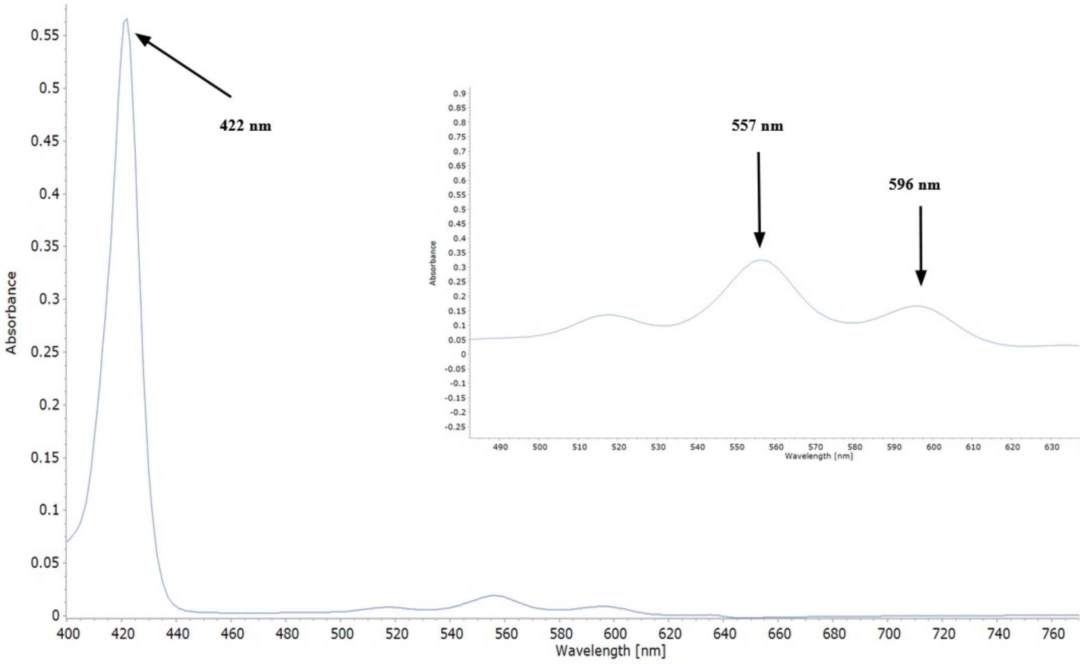

Figure S5 UV-Vis spectra of ZnTCPP

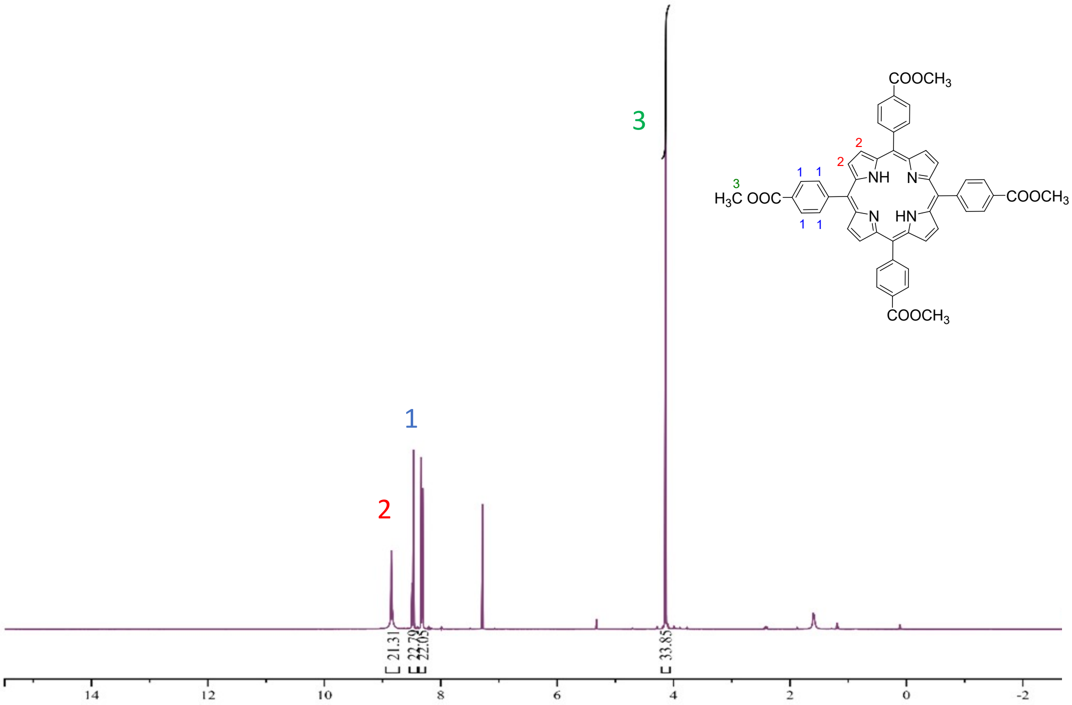

Figure S6 ^{1}H NMR spectrum of TPPCOOMe.

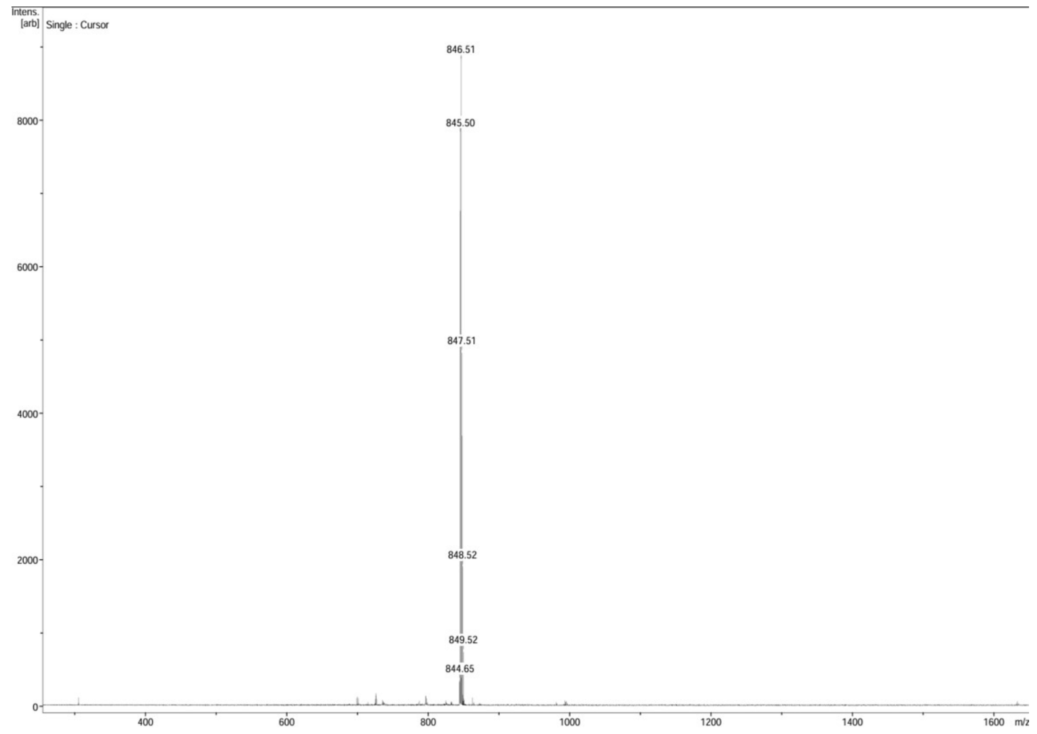

Figure S7 MALDI-TOF-MS spectrum of TPPCOOMe.

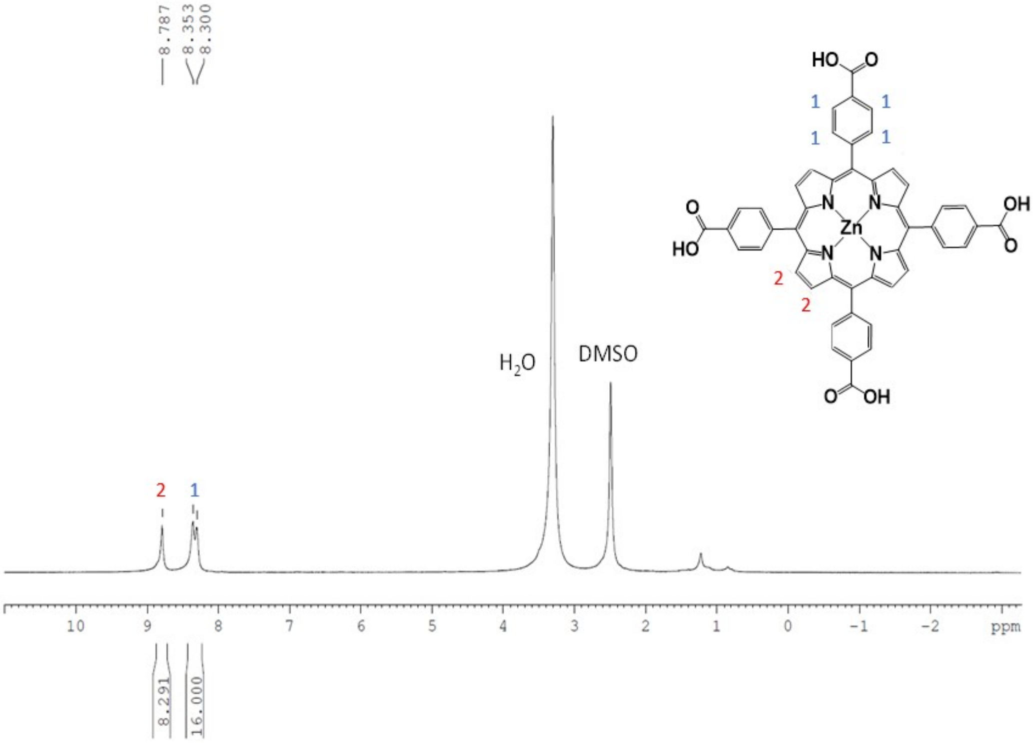

Figure S8 ^{1}H NMR spectrum of ZnTCPP in d6-DMSO.

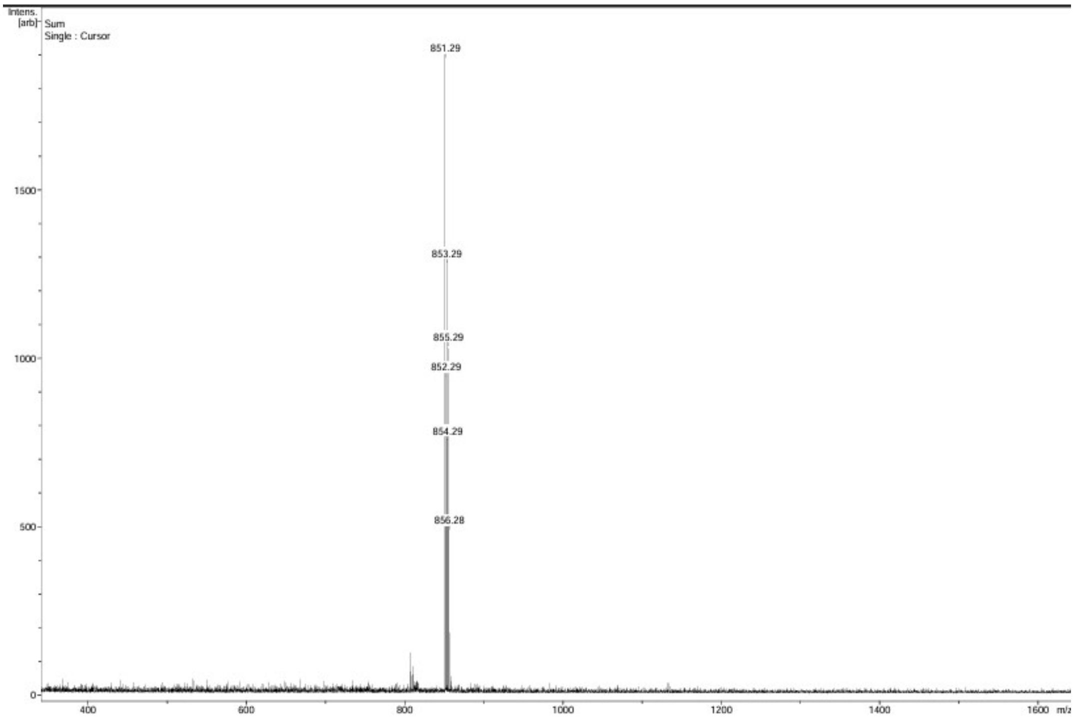

Figure S9 MALDI-TOF-MS spectrum of ZnTCPP.

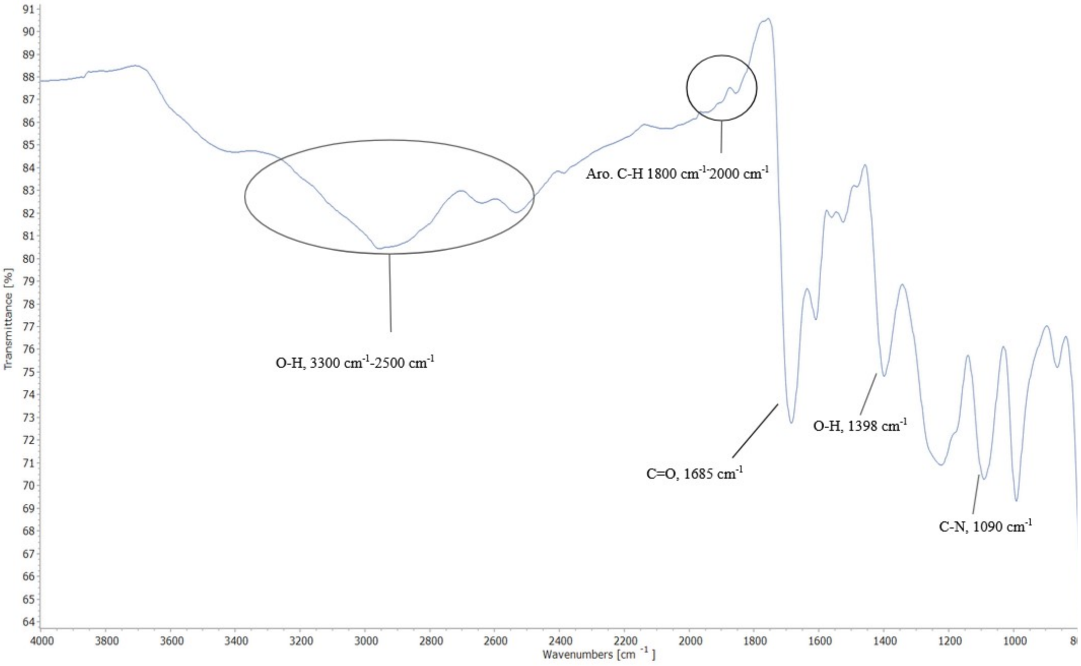

Figure S10 FT-IR spectra of ZnTCPP

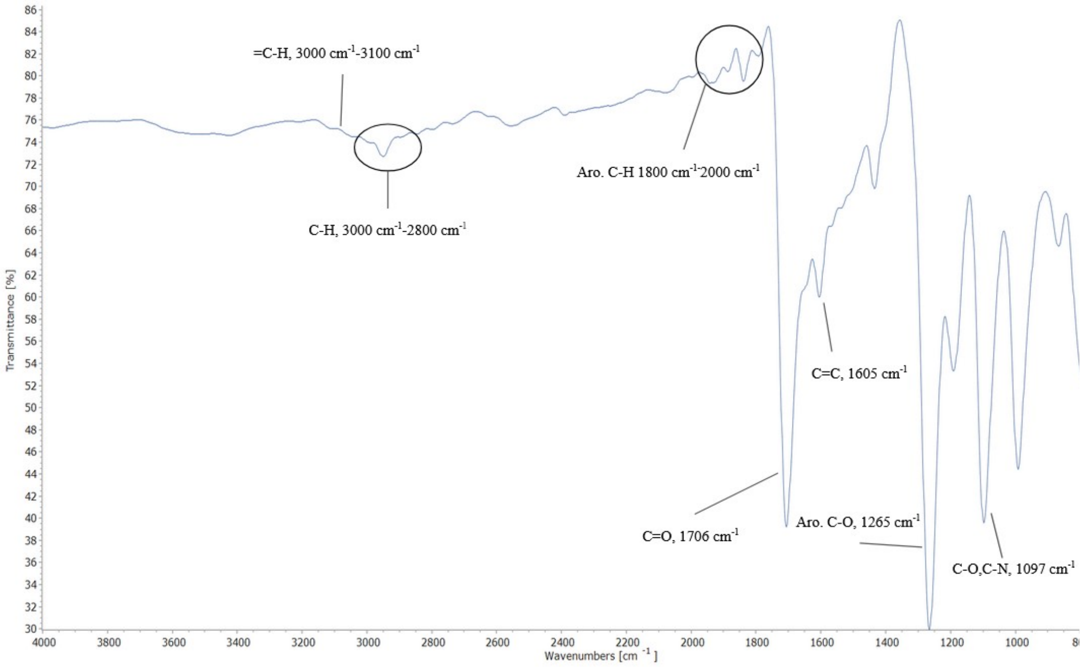

Figure S11 FT-IR spectra of ZnTPPCOOMe

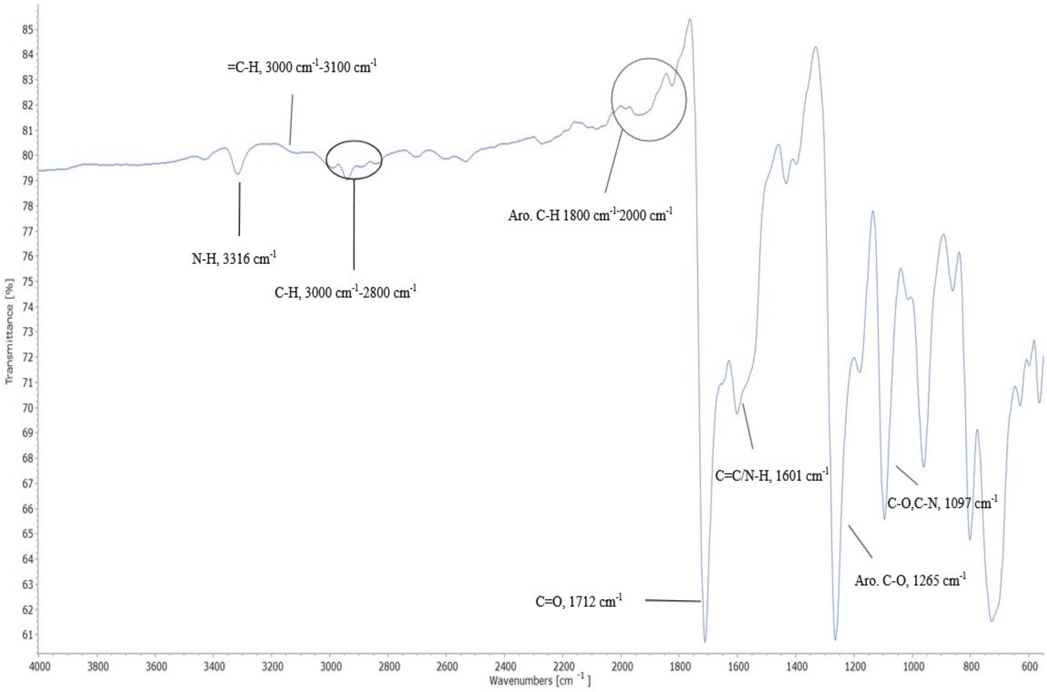

Figure S12 FT-IR spectra of TPPCOOMe

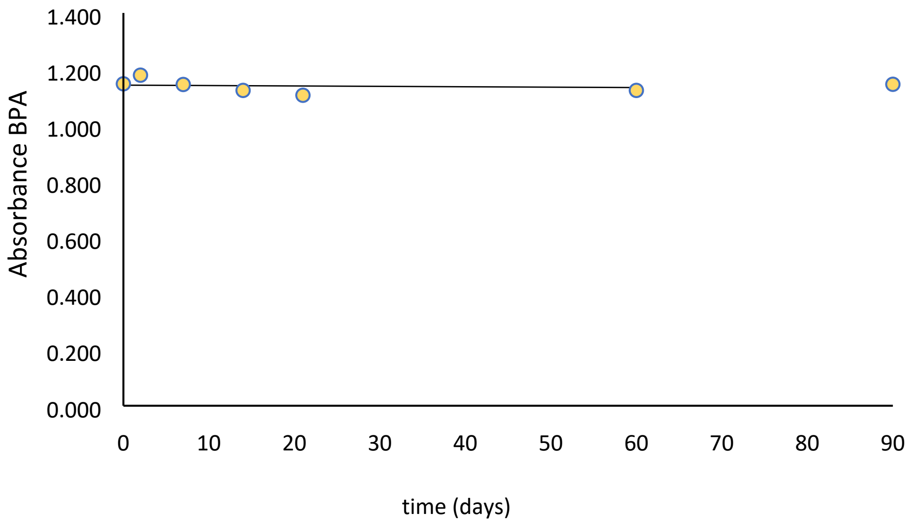

Figure S13 Absorbance measurements of BPA under hydrolysis conditions over a 90-day period, $[BPA]_0$=20 mg L^{-1}

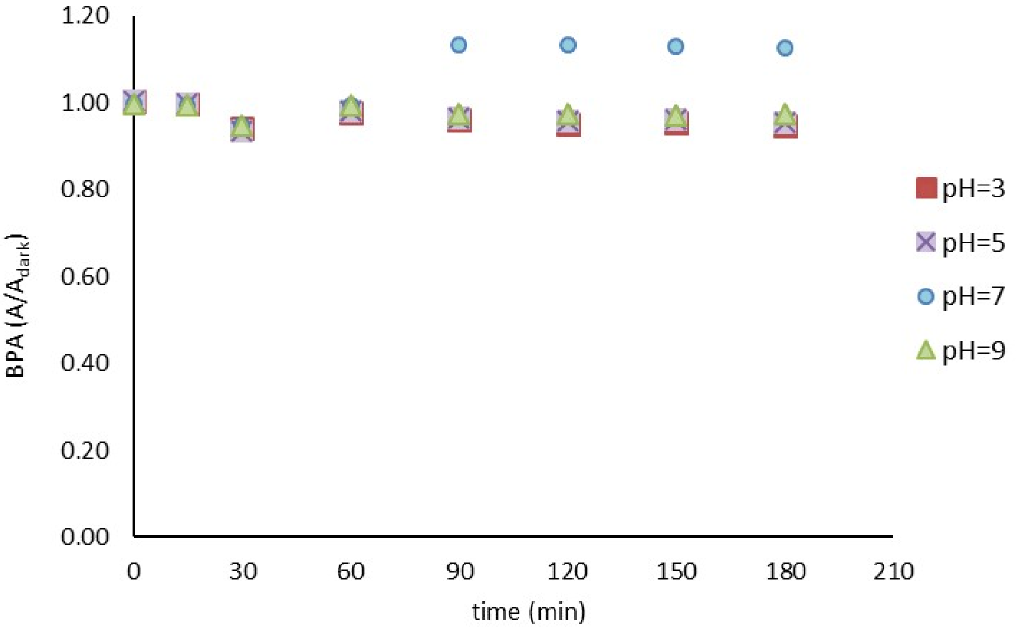

Figure S14 Photolysis of BPA under varying pH conditions (3, 5, 7, and 9), $[BPA]_0$=20 mg L^{-1}
